# Detecting silent whales using seabed fiber-optic cables

**DOI:** 10.1073/pnas.2603077123

**Published:** 2026-06-23

**Authors:** Robin André Rørstadbotnen, Martin Landrø

**Affiliations:** ^a^ Acoustics Group, Department of Electronic Systems, Norwegian University of Science and Technology, Trondheim 7034, Norway; ^b^ Centre for Geophysical Forecasting, Department of Electronic Systems, Norwegian University of Science and Technology, Trondheim 7034, Norway

**Keywords:** distributed acoustic sensing, Arctic, silent sources, pressure fluctuation

## Abstract

Distributed acoustic sensing (DAS) has emerged as a powerful tool for passive whale monitoring, enabling both the detection of vocalizations and the simultaneous tracking of multiple individuals. However, a fundamental limitation of passive acoustic monitoring is that most methods rely on acoustic data, which is only available when whales vocalize. This clearly demonstrates the need for new sensing methods that can detect silent whales. In this paper, we detect hydrodynamic pressure and velocity fields in the low-frequency DAS data induced by a whale’s motion and develop methods to analyze these signals. First, we use ships as proxies to demonstrate and calibrate the proposed method. Then, we show that a simple fluid mechanical model can be adapted to understand how whale swimming can be detected and analyzed using DAS. We detect multiple silent whales simultaneously, estimate their characteristics, and show that whale motion signals decay as one over distance cubed. Moreover, we demonstrate that we can observe hydrodynamic pressure and velocity signals from a cruise ship at 413 m water depth, and up to 550 m from the fiber cable. In comparison, the smaller blue whales can be observed when diving within 40 m of the fiber-optical cable. This sensing method enables an approach to monitoring one of the world’s most endangered species.

## Significance

With present-day acoustic listening technology using seabed fiber-optic cables, it is possible to record and reconstruct the vocalizations of marine mammals. This can be used to track them. However, up to present, it has not been possible to track marine mammals when they are silent. Using the method presented in this paper, we demonstrate that it is possible to detect silent blue whales within a range of 40 m from the fiber. This finding opens up many possibilities for detecting silently moving whales in the water layer.

Due to rapid Arctic climate change, we expect shifts in whale migration patterns and habitat use, concurrent with changes in human activity. Such human activity changes can include the development of new shipping routes through areas previously covered by sea ice (1). Increased human activities in species-rich areas will inevitably disrupt whale habitats (2). Therefore, it is urgent to develop robust, scalable methods to better understand marine mammals’ geographical ranges and habitats.

There are different paths to better understand the impact of climate change on whale behavior. One path is to develop better tracking algorithms that combine new and old data sources, e.g., distributed acoustic sensing (DAS) (3, 4), Permanent Acoustic Monitoring (PAM) networks (5, 6), satellite-based methods (7, 8), tagged whale data (9, 10) and visual observations (11). A second path is to explore datasets further and discover new, valuable signatures. In this work, we follow the second path. The signature identified in this work is observed in the low-frequency component of ship and whale signals recorded by DAS. These signals are interpreted as a translating hydrodynamic field, consisting of both pressure variations and the associated particle velocity field generated by the displacement of water as ships or whales move through the ocean. As these components are intrinsically linked, we refer to their combined effect as a hydrodynamic pressure field throughout this work. This means that no acoustic signal is involved, and we can “listen” to silent whales.

DAS is a fiber-optic measurement technique that uses coherent Rayleigh backscattering to record strain along the fiber with high spatial and temporal resolution. The method is based on phase measurements of the Rayleigh backscattering to capture the strain field along a fiber at timescales controlled by the kilohertz laser pulse repetition rate (12). DAS has traditionally been used to study high-frequency signals (4, 13, 14). More recent work has demonstrated that it can also resolve significantly lower-frequency variations (15–17).

In the marine environment, low-frequency strain variations (0.01 to 0.1 Hz) may arise from a range of physical mechanisms, including hydrodynamic processes associated with moving objects. The displacement of water generated by moving whales and ships induces a moving hydrodynamic pressure field that extends through the water column, dynamically loading and unloading the seafloor. This varying pressure field causes the seafloor sediments to deform both vertically and laterally. When the fiber is buried in sediments, the DAS system primarily records the resulting lateral deformation of the surrounding material. Hydrodynamic pressure fluctuations produced by ocean waves have already been extensively studied (18–21). However, for more detailed characterization of pressure signals, dedicated pressure sensors are often employed. For example, Stenvold et al. (22) demonstrated that high-precision seabed pressure sensors can detect subtle pressure variations, highlighting their potential for hydrocarbon reservoir monitoring. Related studies have also explored acoustic and hydrodynamic signals generated by moving objects. Hegna (23) investigated how acoustic wavefields generated by moving vessels, recorded by towed streamers or ocean-bottom sensors, can be used to image the subsurface. Another study by Werner and Landrø (24) investigated the hydrodynamic pressure field generated by a buoy moving through the water column, while Scarpa et al. (25) used pressure sensors to measure ship wakes in Venice, Italy, and assess their impact on the lagoon. The pressure field associated with ship-generated Kelvin wakes typically contains frequencies between 0.1 and 0.4 Hz (26–28). Buisman and Thiem (29) investigate hydrodynamic signals from ships recorded with different DAS configurations in both very shallow and deeper water settings, down to 58 m. In very shallow water, they report low-frequency signals attributed to ship wakes, including contributions from bow and stern waves within the Kelvin wake system. In deeper water, they observe even lower-frequency signals, which they describe more generally as “ship-induced water waves,” without drawing a firm conclusion on whether these arise from the Kelvin wake or from other displacement-driven processes. In this study, we present observations of ship- and whale-generated signals from a DAS installation in Svalbard, where the recorded signatures fall within the same low-frequency band as those observed in the deeper-water setting of Buisman and Thiem. Building on these earlier observations, we introduce a theoretical framework that explains the origin of these signals. We show that the low-frequency response is consistent with translating hydrodynamic pressure fields generated by the displacement of water by moving ships and other large bodies such as whales. This interpretation is developed in detail in the following sections (see also *SI Appendix*, section 2).

DAS is topical across many scientific fields, including geophysics and marine biology (4, 30, 31). It has been labeled a ‘game-changer’ across many disciplines, especially in remote sensing and ocean monitoring. There are different reasons for this. 1) The instrumentation is placed on land or on a platform; 2) No DAS sensor components are exposed to the harsh underwater environment; 3) DAS can use the existing telecommunication infrastructure; 4) The interrogator signal can reach up to 140 km (12). New steps have been taken to increase the DAS range to exceed 1,000 km (32, 33), opening new possibilities for monitoring ships and whales over vast areas.

Most studies on whales using DAS and PAM have so far focused on the mammals’ acoustic signatures. The hydrodynamic pressure field signatures are independent of acoustic pressure signatures and can track and monitor silent whales as long as they are sufficiently close to the fiber.

## Results

### Experiment Overview.

**Fig. 1.**
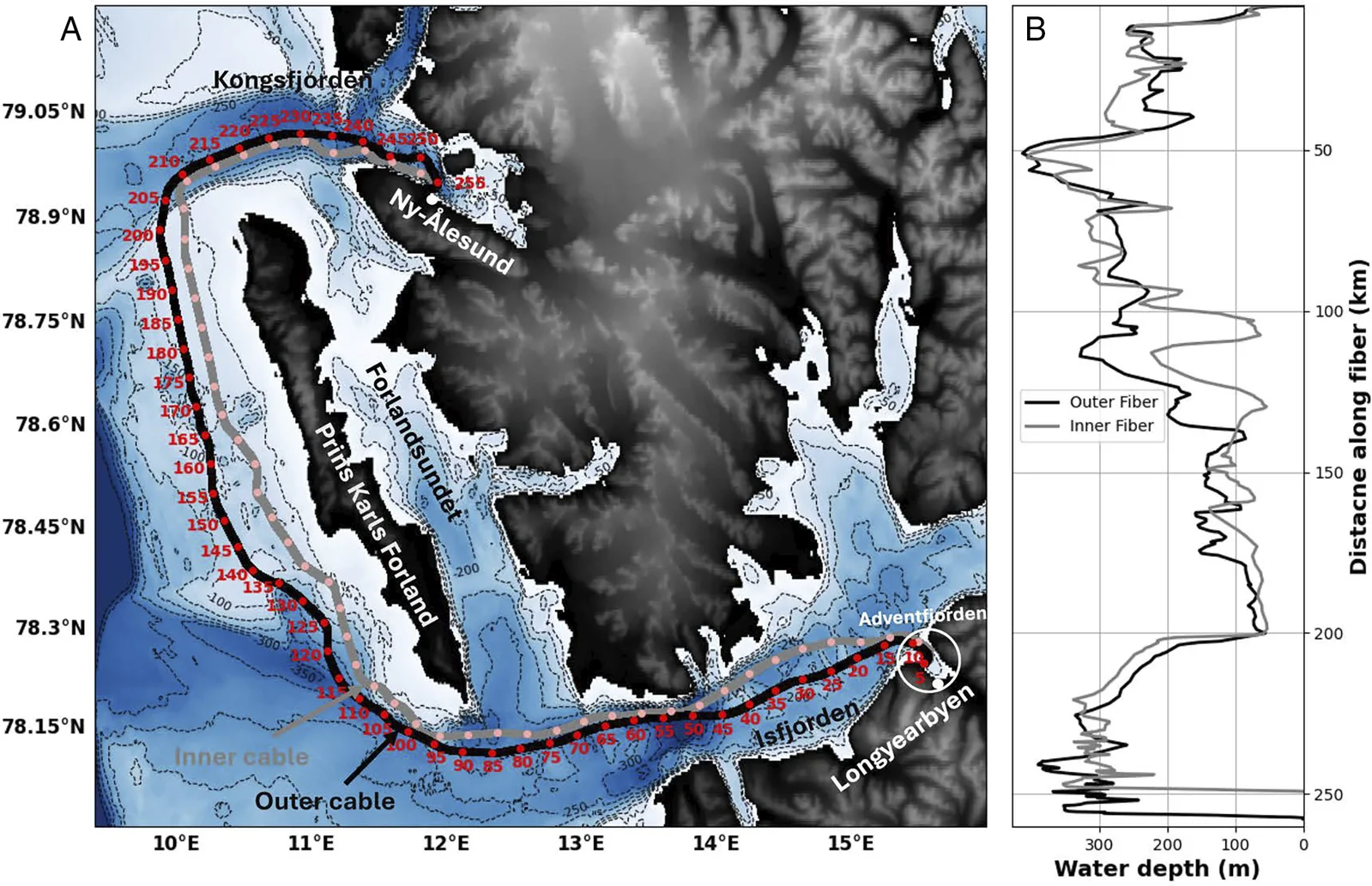
The fiber cables connecting Lonbyearbyen and Ny-Ålesund. (*A*) The fiber lay-out. The white circle indicates the study area used in subsequent figures. The numbers indicate the distances from the interrogator unit in Longyearbyen in km. (*B*) Water depth to fiber.

During the DAS experiment on Svalbard in 2022, four interrogator units (IU) were installed. Two units were placed in Longyearbyen, and two in Ny-Ålesund. Upon the conclusion of the acquisition, three of the IUs had been shipped back to mainland Norway, while the last has been recording in Ny-Ålesund since. In March 2025, another IU was installed in Longyearbyen and has been recording since. This work focuses on data recorded by the instrument in Longyearbyen, recording on the outer fiber (Fig. 1). However, the complete data-set was used to analyze how coupling affects the detectability of hydrodynamic pressure field signatures and how the signal characteristics change with increasing source-receiver distance.

The interrogators were connected to standard fibers within two different submarine cables. The total length of each cable is ∼260 km, where each interrogator acquires high-quality data for 80 to 90 km, after which the noise floor rapidly increases due to the low amount of laser energy left in the fiber cable. Due to this distance limitation, we needed four interrogators to cover the complete length of the FO cables. A gauge length of 8.16 m and a spatial sampling of 4.08 m were typically used. For more information, see Rørstadbotnen et al. (34).

The water depth along the FO cable is depicted in Fig. 1*B*. The cables enter Adventfjorden at 5.1 km, where the water depth rapidly drops to 70 to 80 m. In this study, we focus on the shallow-water region, while the deeper waters in Isfjorden and Kongsfjorden are used to study various signal characteristics. The fiber cables are buried in the sediments in these fjords. To the west of Prins Karls Forland, the fiber is unburied, with water depths similar to those in Adventfjorden. This shelf area is used to observe how coupling affects recordings of the acoustic and hydrodynamic pressure field signals.

### Ship Signatures in the Time Domain.

Ships are observed in the DAS data year-round, and the AIS is available to confirm which ship is sailing above the fiber and at what time. We investigate four ships of different sizes, observed at roughly the same water depth (70 to 80 m) and sailing speed. It is eminently clear from the DAS data that the observed signal amplitude scales with the size of the ship (*SI Appendix*, Fig. S3). The smallest ship is Kvitungen, and its amplitude is barely above the noise floor. Helmer Hansen (HH) is 2.3 times bigger and generates stronger acoustic signals. The icebreaker ODEN is 4.0 times larger than HH, as evidenced by higher strain levels. The largest ship, LCC, produces acoustic amplitudes similar to ODEN. Moreover, we use AIS tracks to understand data signatures better. For example, LCC and ODEN are closer to the fiber over a shorter time than HH, which explains their more localized acoustic signature. HH, on the other hand, crosses the fiber at 6.15 km and sails along the fiber for 1 to 2 km, thereby picked up on more channels.

**Fig. 2.**
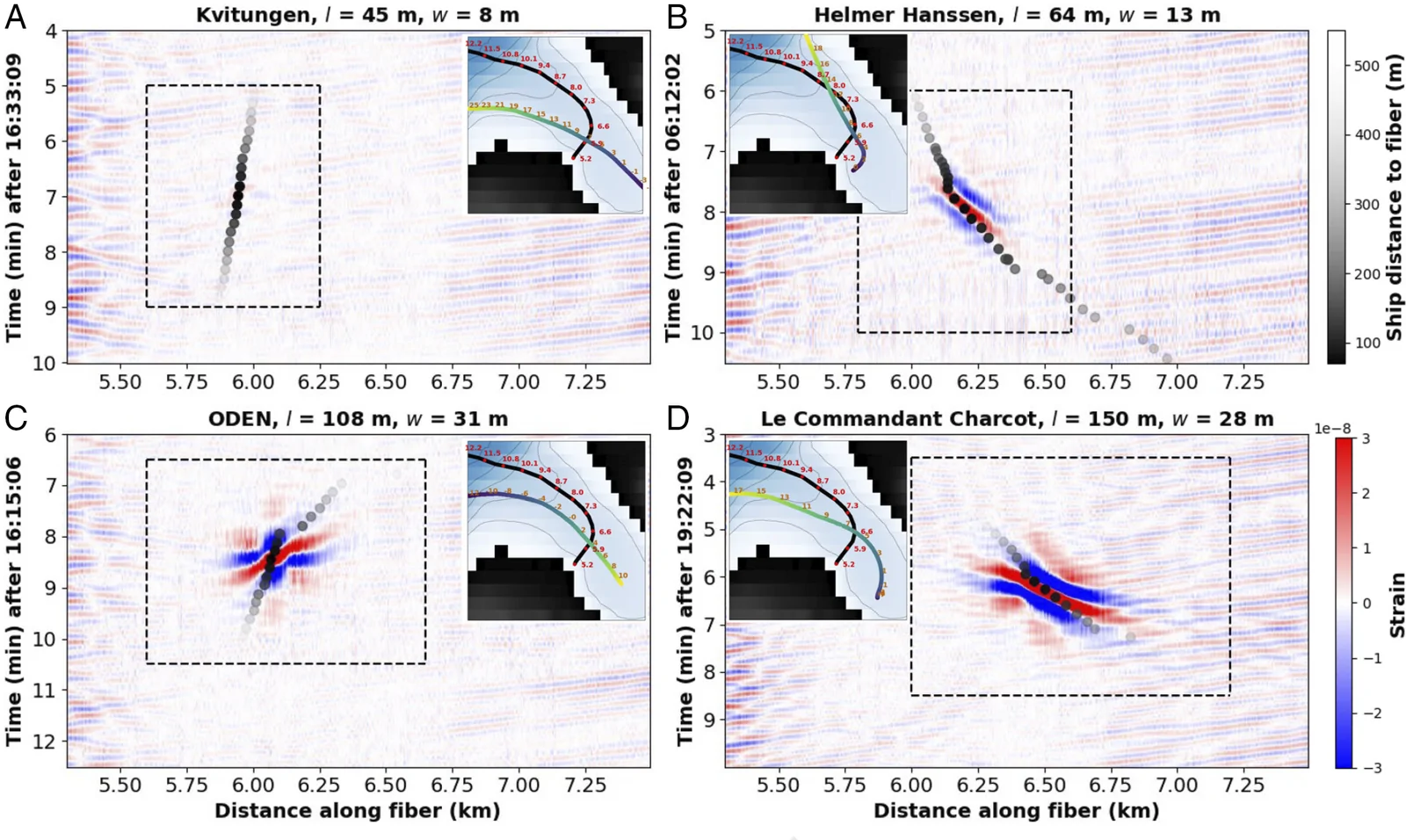
Low-frequency signatures between 0.008 and 0.07 Hz of four ships of different sizes in the time-distance domain. (*A*) The smallest ship, Kvitungen, with a length of 45 m and a beam width of 8 m. (*B*) The ship Helmer Hanssen with length 64 m and beam 13 m. (*C*) The icebreaker ODEN with length 108 m and beam 31 m. (*D*) The largest ship, the cruise ship Le Commandant Charcot (LCC), length 150 m and beam 28 m. In all figures, the distance from the automatic identification system (AIS) position to the nearest virtual DAS channel is indicated by gray circles, with transparency decreasing with decreasing distance. The *Insets* show the AIS track relative to the fiber cable in Adventfjorden (see white circle in Fig. 1) and dashed rectangles indicate the windows used for frequency and frequency-wavenumber analysis.

The recorded acoustic signatures show that the signal strength scales with the size of the ship generating it. When a ship moves across the ocean surface, it pushes water ahead of it and draws water along in its wake. This motion generates a hydrodynamic pressure field that extends downward through the water column. As this pressure field reaches the seafloor, it translates into strain that can be observed in the DAS data. Fig. 2 displays the low-frequency data at the time the four ships cross the fiber, focusing on frequencies between 0.008 and 0.07 Hz. The hydrodynamic pressure field signal is similar to the acoustic signatures in that the amplitudes scale with the ship size and the distance from the ship to the fiber cable. Kvitungen’s signature (Fig. 2*A*) is barely observable above the noise floor, whereas HH (Fig. 2*B*), ODEN (Fig. 2*C*), and LCC (Fig. 2*D*) have significant signal-to-noise ratios. The main difference from the acoustic pressure signatures is that the detection range is smaller. By comparing the acoustic ship data in *SI Appendix*, Fig. S3 to Fig. 2, we see that the main low-frequency energy appears at the same time as the apex of acoustic pressure signals generated by the same ship. This is also evident in the data, where LCC is recorded over a longer buried fiber segment and when the fiber is unburied (*SI Appendix*, section S4).

### Time-Domain Model of a Moving Hydrodynamic Particle Velocity Field.

**Fig. 3.**
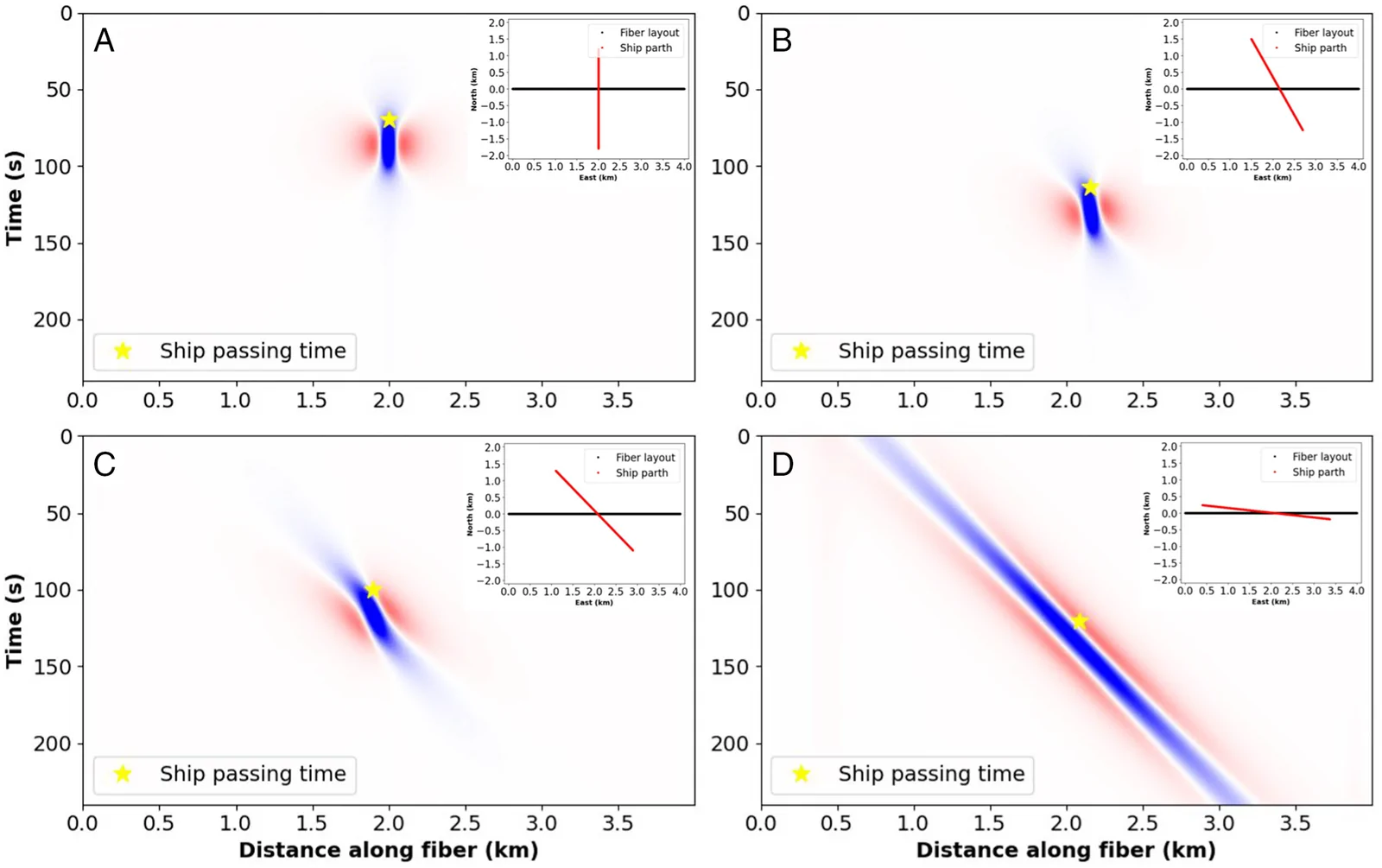
Hydrodynamic modeling in strain units. Modeled response along the fiber using a sinusoidal wavelet as source and the size of LCC. (*A*) Ship crosses the fiber perfectly perpendicular (90^°^) to the fiber. (*B*) Ship crosses at 78.1^°^. (*C*) Ship crosses at 53.1^°^. (*D*) Ship crosses at 8.1^°^. Note that ship paths relative to the fiber cable are depicted in the *Insets*.

To better understand the low-frequency signatures observed in Fig. 2, we develop a fluid-flow model based on Rayleigh’s formulation (35) to describe how a moving hydrodynamic particle velocity field gives rise to signals recorded along a linear array (see *Materials and Methods* for details). The obtained hydrodynamical particle velocity field using Eq. **6** is subsequently converted to strain units by Eqs. **7** and **8**. The modeling results are shown in Fig. 3, where a ship with dimensions similar to LCC crosses the fiber at four different angles: 90^°^ (Fig. 3*A*), 78.5^°^ (Fig. 3*B*), 53.1^°^ (Fig. 3*C*), and 8.1^°^ (Fig. 3*D*). The main difference between the modeling set-up and the Svalbard fiber cable is that the linear array used in the modeling is straight, whereas the Svalbard fiber has curved segments. The modeling for a ship crossing the fiber perpendicularly predicts a symmetric response without polarity reversal. As the sailing direction becomes more parallel to the fiber, the signal can be tracked over longer distances, reaching several kilometers for the 8.1^°^ case.

The corresponding frequency-wavenumber (*f*–*κ*) representations are shown in *SI Appendix*, Fig. S8. These demonstrate that the apparent *f*–*κ* signature depends strongly on the angle between the vessel trajectory and the fiber segment used. When the vessel is sailing nearly parallel to the fiber, the energy collapses onto a line consistent with f=Vκ, as expected from the Fourier transform of a translating signal (Eq. **5**). This allows for a reliable estimation of the sailing speed used in the modeling. In contrast, for larger crossing angles, the response becomes increasingly smeared in the *f*–*κ* domain. Extracting the sailing speed is most challenging for perpendicular crossings. However, as the trajectory becomes more parallel to the fiber, the signal becomes progressively more coherent.

### Ship Signatures in the Frequency-Wavenumber Domain.

**Fig. 4.**
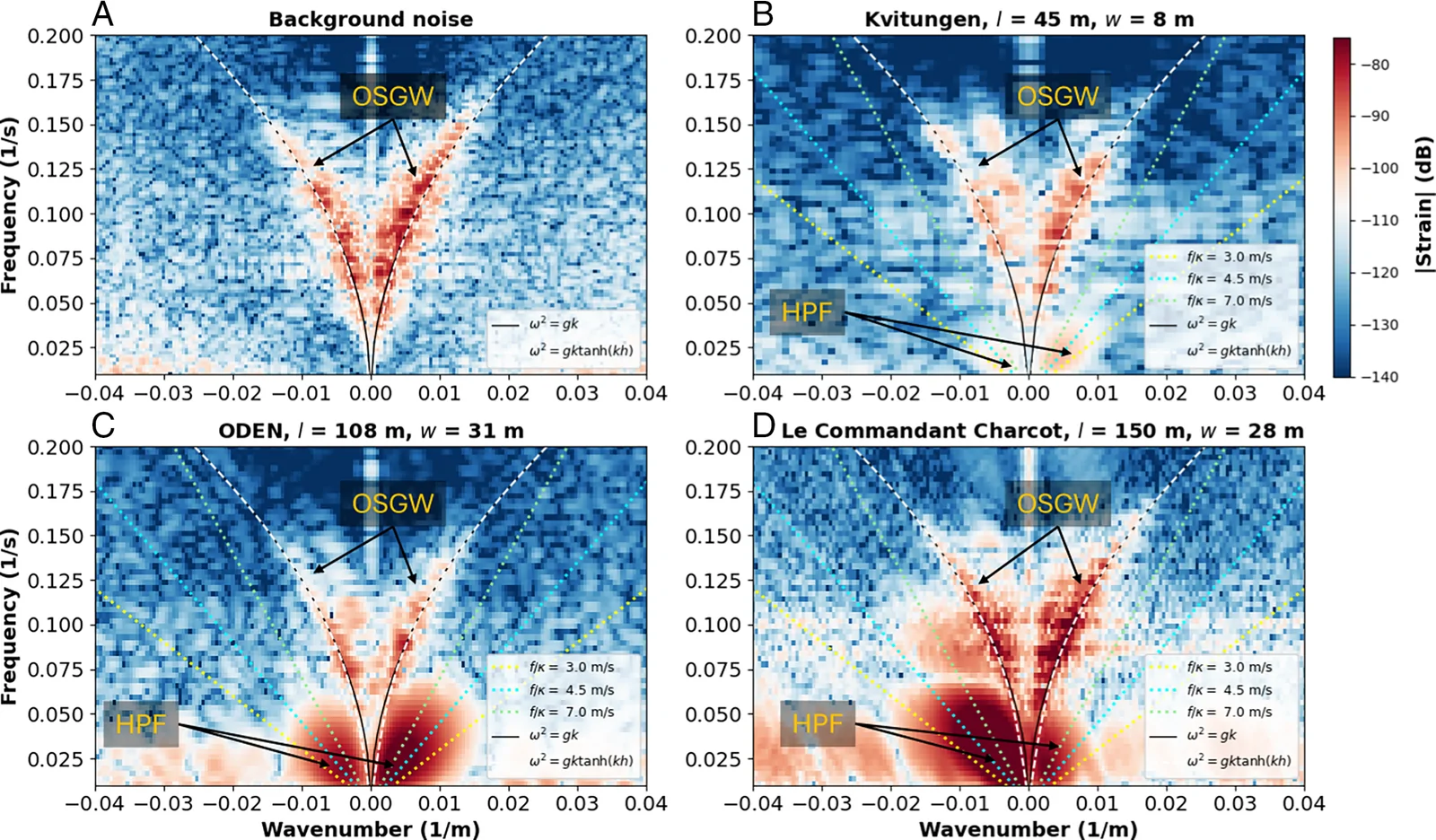
Low-frequency signatures of four ships of different sizes in the frequency-wavenumber domain. Ocean surface gravity waves (OSGW) and the hydrodynamic pressure field (HPF) are marked by black arrows. The hydrodynamic pressure field occurs for lower frequencies and has a linear appearance (green, yellow and cyan lines) compared to the curved appearance (white and black lines) of OSGW in the *f*–*κ* plot.

After establishing the time-domain signatures of acoustic and hydrodynamic pressure field signals produced by moving ships, we now examine their temporal and spatial frequency content. Fig. 4 shows the *f*–*κ* domain for frequencies below 1 Hz (see *SI Appendix*, section S6 for higher frequencies). Fig. 4*A* shows the background noise, where the dispersive arrival of the ocean surface gravity waves (OSGW) is the only signal present. When the ship signals are included, the results shown in Fig. 4*B*–*D* exhibit additional arrivals at frequencies below the OSGW. These are nondispersive and scale with ship size. ODEN crosses the fiber nearly perpendicularly and has almost equal energy at positive and negative wavenumbers. Conversely, Kvitungen and LCC exhibit energy primarily at negative wavenumbers, consistent with their AIS tracks. The signatures below the OSGW are interpreted as the hydrodynamic pressure field generated by moving ships. Note that the low-frequency signal shown in Fig. 2 is not associated with the Kelvin wake, which typically contains higher frequency components (25, 28). Moreover, if we interpreted our data within Kelvin wake theory, the observed low frequencies would require unrealistically high vessel velocities (e.g., ∼65 to 70 m/s for ODEN and LCC), well above those observed in this study. These speeds even exceed the phase velocity at which ocean surface gravity waves propagate freely once detached from the ship hull (∼27 m/s for a water depth of 76 m). Additionally, Kelvin wake waves follow a dispersive gravity–wave relation and therefore produce a curved signature in the *f*–*κ* domain. In contrast, the observed low-frequency signal shows a linear *f*–*κ* relationship consistent with a translating hydrodynamic pressure field, indicating a different physical origin (*SI Appendix*, section 2). Finally, the pressure field associated with Kelvin wake waves decays exponentially with depth (*SI Appendix*, section S7). Given their relatively short wavelengths (∼13 to 32 m), these signals are strongly attenuated with depth, and they are therefore not expected to be detectable at the water depths studied here.

If we assume that the ship first pushes the water over its entire length, followed by an opposite backflow of similar extent, the dominant frequency for one sequence can be expressed as f=V/2L, where *V* is the speed of the ship and *L* is the length of the ship. In our simple model (*Materials and Methods*), we approximate this behavior as an oscillatory movement, similar to an air bubble expanding and collapsing in water (35–37). We extract the dominant frequency for each DAS channel containing low-frequency signals to estimate ship sailing and whale swimming speeds (*SI Appendix*, section S8). We do this first for HH, ODEN, and LCC and estimate their respective speeds to be 3.0 ± 0.5 m/s, 4.6 ± 0.8 m/s, and 7.2 ± 0.9 m/s (see *Materials and Methods* for uncertainty computations). The obtained values are compared to speeds derived from AIS tracks (Table 1). For ODEN and LCC, the speeds from AIS are within one SD of those from low-frequency signatures. For HH, the speed is underestimated, which we attribute to the vessel accelerating from 2 to 3 m/s to the given velocity of about 4.5 m/s shortly before our low-frequency recording. The origin of this discrepancy is unclear but may be related to timing inaccuracies in the AIS data, limitations of the current methodology in capturing vessel acceleration, or uncertainties in the identification of the dominant low-frequency peak.

**Table 1.** Observed (obs) and estimated (est) values for blue whales and ships

Name	*L* (m)	$f_{\text{obs}}$ (Hz)	$V_{\text{est}}$ (m/s)	$A_{\text{max}}$ (n$\epsilon_{x}$)	$z_{\text{est}}$ (m)	$L_{\text{est}}$ (m)
Blue whale 1	26* ± 1	0.1015 ± 0.017	5.3 ± 0.9	167.3 ± 61.1	33.5 ± 5.7	38.9****± 5.88
Blue whale 2	26* ± 1	0.0786 ± 0.011	4.1 ± 0.5	364.4 ± 149.9	23.5 ± 5.3	21.2 ± 2.97
Blue whale 3	26* ± 1	0.0618 ± 0.023	3.2 ± 0.9	48.8 ± 6.4	40.9 ± 6.8	8.98 ± 1.25
Blue whale 4	26* ± 1	0.0665 ± 0.009	3.5 ± 0.5	210.5 ± 46.1	26.4 ± 3.1	32.8****± 2.99
Blue whale 5	26* ± 1	0.0626 ± 0.015	3.2 ± 1.1	76.3 ± 10.7	35.4 ± 4.0	25.0 ± 4.02
Blue whale 6	26* ± 1	0.0739 ± 0.009	3.9 ± 0.5	130.0 ± 39.2	32.5 ± 4.6	20.1 ± 2.31
Blue whale 7	26* ± 1	0.0690 ± 0.009	3.5 ± 0.6	397.5 ± 106.2	21.7 ± 2.7	37.7****± 4.28
Blue whale 8	26* ± 1	0.0566 ± 0.006	2.8 ± 0.4	87.6 ± 29.5	33.7 ± 5.5	27.1 ± 3.73
Blue whale 9	26* ± 1	0.0677 ± 0.015	3.5 ± 0.8	251.8 ± 70.4	24.9 ± 3.6	18.6 ± 2.3
Blue whale 10–12	26* ± 1	0.0807 ± 0.020**	4.2 ± 0.6	206.8 ± 85.9	28.6 ± 6.4	23.0 ± 3.97
Blue whale 13	26* ± 1	0.0462 ± 0.002	2.4 ± 0.2	22.8 ± 2.6	35.0 ± 2.7	21.8 ± 3.7
HH	64	0.0232 ± 0.003	3.0 ± 0.5 (4.5)	37.3 ± 5.6	63.6 ± 5.3*** (76)	68.9 ± 5.76
ODEN	108	0.0214 ± 0.004	4.6 ± 0.8 (4.5)	106.2 ± 24.4	74.7 ± 8.7 (76)	94.5 ± 8.38
LCC	150	0.0246 ± 0.003	7.2 ± 0.9 (7.0)	274.3 ± 35.2	77.9 ± 6.0 (77)	150.0 ± 0.0

**Fig. 5.**
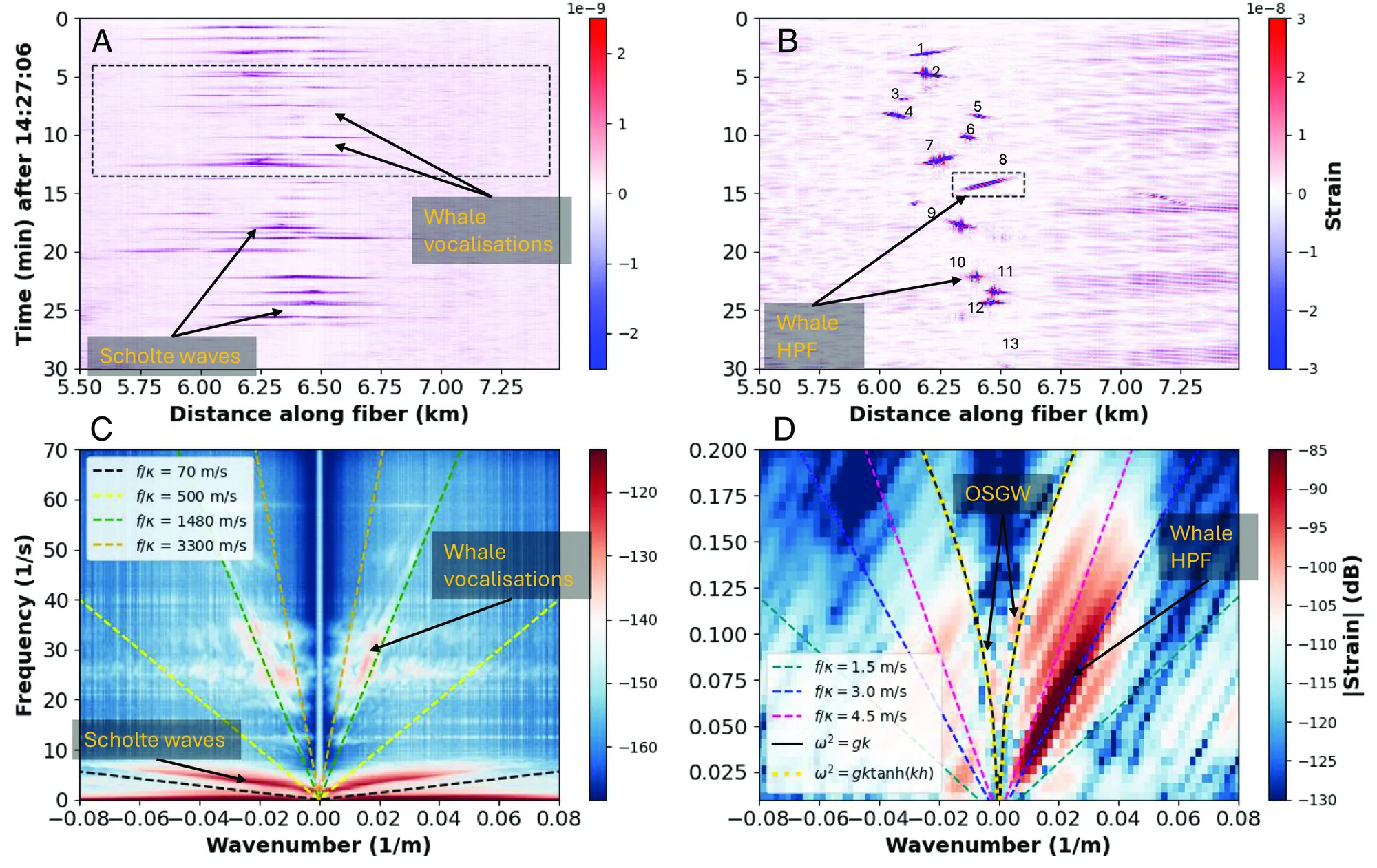
Blue whale signatures in different frequency bands and different domains. (*A*) High-frequency data (5 to 70 Hz data) in *t-x* domain. (*B*) Low-frequency data (0.008 to 0.22 Hz data) in *t-x* domain. (*C*) Frequency content with linear scale overlaid by dashed lines showing various propagating speeds. The data window used is indicated as dashed square in (*A*). (*D*) Frequency content focusing on data between 0.01 and 0.2 Hz. The data window used is indicated as dashed square in (*A*). The annotations show our interpretation of the signal. In (*C*) we identify the whale vocalizations as the fast propagating waves and Scholte waves as the slow. In (*D*) we identify the ocean surface gravity waves (OSGW) as the curved dispersive wave and the linear ridge as the hydrodynamic pressure field generated (HPF) by the moving whale.

*Assumed values (40), **Three overlapping whale signals; a single frequency is stated for all signals, ***Using the correct velocity gives a correct water depth of 73.0 ± 5.2 m, ****Abnormally big animal.Summary of sizes of blue whales, HH, ODEN and LCC, their observed frequencies and amplitudes, estimated speed using Eq. **21**), estimated depth using Eq. **4** and estimated lengths from Eq. **15**. Uncertainty is given as one SD (*Materials and Methods*). The $f_{\text{obs}}$ values are the frequency with the maximum amplitude for each channel. $A_{\text{max}}$ is given as the mean and SD of the top 1% of the amplitudes. See Fig. 5 for the numbering of the blue whales. Measured vessel speeds for the ships (from AIS) are given in parentheses in column 4, and corresponding measured distances to the fiber is given in parentheses in column 6.

The dominant frequency components are also extracted from signals attributed to whales. All extracted frequencies are higher than those observed for ships and yield estimated speeds between 2.8 and 3.8 m/s, with one outlier at 5.1 m/s (see Table 1 for individual estimates). These values are consistent with typical transit swimming speeds for blue whales but are higher than those expected for feeding behavior (38, 39).

The estimated speeds agree with the apparent slopes in Figs. 2 and 5*D*. The low-frequency energy appears slightly smeared in the *f*–*κ* domain, likely due to the slight curvature of the fiber and the inherently limited frequency–wavenumber resolution at these low frequencies. In addition, the modeling results (*SI Appendix*, Fig. S8) show that signals from vessels crossing the fiber at large angles are expected to be more smeared in the *f*–*κ* domain, consistent with the observations. For both Kvitungen and ODEN the main energy is centered at 4.5 m/s agreeing with estimates and AIS-derived speeds. For LCC there is more energy at higher apparent velocities agreeing with its higher sail speed as it moves over the fiber. For the whale example in Fig. 5*D* we see energy at lower velocities primarly between 3 and 4.5 m/s. This is slightly higher than the speed estimate for this whale, which is 2.8 ± 0.4 m/s.

### The Acoustical, Geophysical, and Hydrodynamical Signatures Generated by Blue Whales.

Whales have been observed in the Svalbard DAS data since the first test in 2020, where several different whale calls were observed (3, 41). Further analyses were carried out using four interrogators, where fin whales were tracked for 5 h (34). These fin whale calls were only a subset of all whale calls recorded during this experiment. On 24 August 2022, several calls were observed together with Scholte waves (seafloor interface waves) and hydrodynamic pressure field signals (Fig. 5). By analyzing spectrograms, we interpret the calls as downsweeps from blue whales (*SI Appendix*, section S9). While previous work using this dataset has focused on fin whales due to their frequent vocalizations (34, 42), the signals analyzed here are dominated by sweeps starting around 60 Hz and ending near 20 Hz, consistent with blue whale calls (3). We therefore focus our analysis on a time window containing these blue whale calls and their associated low-frequency signatures. This choice is also motivated by two additional factors: 1) Blue whales are the largest baleen whales and are therefore expected to generate the strongest hydrodynamic pressure field signals, making them the most likely to be detected by DAS; 2) Three small vessels were present near the fiber, none of which overlap with the low-frequency data, and all at distances exceeding the detection range estimated from LCC. These vessels are therefore not expected to contribute to the observed signals (*SI Appendix*, section S10). This allows us to attribute the observed low-frequency signals to the blue whales rather than ship traffic.

To better understand the nature of the signals observed in the DAS data, we study the whale-induced signals in more detail. Fig. 5 shows the *t*–*x* and *f*–*κ* representations of these events. Fig. 5*A* shows both the acoustic and geophysical signatures. Within the first 10 min, the blue whale downsweeps appear as faint horizontal lines. After approximately 10 min, the vocalizations stop, and Scholte waves primarily dominate the data. Fig. 5*B* focuses on the low-frequency band where the hydrodynamic pressure field signals are recorded (*SI Appendix*, section S11). From this panel, we select 13 labeled signatures for further analysis. Fig. 5 *C* and *D* show the corresponding *f*–*κ* representations for the high- and low-frequency bands. Fig. 5*C* highlights the acoustic vocalizations and the Scholte waves. It is worth noting that no ship signatures are present, as can be observed in the *f*–*κ* domain representation of the various ship signals given in *SI Appendix*, Fig. S9. Fig. 5*D* shows the low-frequency part in the *f*–*κ* domain. The observed signatures are similar to those of the ships shown in Fig. 4, but with higher frequency and wavenumber content. The dominant frequency in one example (whale 8 in Table 1) is 0.057 Hz, which, according to Kelvin wake theory, corresponds to a minimum velocity of 20.8 m/s. The highest frequency observed among the 13 whale examples is 0.1 Hz (whale 1), corresponding to a minimum velocity of 15.6 m/s. These velocities are significantly higher than expected whale swimming speeds and also exceed the maximum vessel speeds observed in this study (∼10 m/s). Thus, even when the frequencies approach the Kelvin wake range, the corresponding velocities remain unlikely. Furthermore, these low-frequency signals are nondispersive, consistent with a translating hydrodynamic pressure field rather than dispersive surface gravity waves (e.g., associated with Kelvin wakes).

### Estimation of Object Characteristics.

We use the theory outlined in *Materials and Methods* to estimate the whale’s depth when generating the hydrodynamic pressure field signal. This is done by comparing a signal generated by LCC, at a known water depth, to one generated by a blue whale at an unknown water depth. The water depth is computed using Eq. **20**. All other values are either known, have been computed or can be extracted from the data. Plugging in the known or estimated values, we compute the water depth for a subset of the low-frequency examples in Fig. 5. The results are summarized in Table 1, indicating that all whales generating low-frequency signals are within 40 m of the fiber. The estimated depths are deeper than the normal vocalization depths of blue whales (≤35 m), but not uncommon for nonvocalizing, diving, blue whales (6, 43, 44).

We calibrate our method using signals from HH, ODEN, and LCC. A key difference in Eq. **20** between whales and ships is that whales are fully submerged, whereas ships are surface-bound and only partially submerged. In the current version of the equation, we have multiplied the whale amplitude by two. The difference compared to surface-bound vessels is accounted for by this factor of two, reflecting the approximate symmetry of the hydrodynamic pressure field around a submerged object compared to the reduced symmetry for a surface-bound vessel. We interpret this factor in terms of an equivalent source geometry: a fully submerged object generates a more symmetric pressure field, while a surface-bound ship produces a truncated field due to the air–water interface, which limits the downward extent of the pressure field. If the ship were fully submerged, a similar factor of two would be expected. This treatment represents a first-order approximation, and further work is required to quantify this factor more accurately. Comparing ODEN and LCC, we find that the estimated water depths agree well with the known bathymetry along their tracks. For HH, we underestimate the water depth by 13 m, but as noted above, we also underestimate the sailing speed by 1.5 m/s. Using the AIS-derived speed, we find the correct water depth (Table 1).

Finally, we estimate the lengths of the whales using Eq. **15**. To obtain more reliable estimates, we first calibrate the model using LCC data, for which the length is known. This is done by deriving a scaling factor from LCC observations. The scaling factor is initially tested on data from ODEN and HH, yielding reasonable length estimates (Table 1). We then apply the same scaling to the whale data to estimate their lengths. The resulting estimates range from 9 to 39 m. The three estimates exceeding 30 m are considered uncertain, as they are larger than the typical size of a large blue whale. In addition, the 9.0 m estimate appears unrealistically small and is associated with a depth of 42 m above the fiber, which is unlikely. The remaining length estimates, together with their corresponding depths, are considered reasonable.

**Fig. 6.**
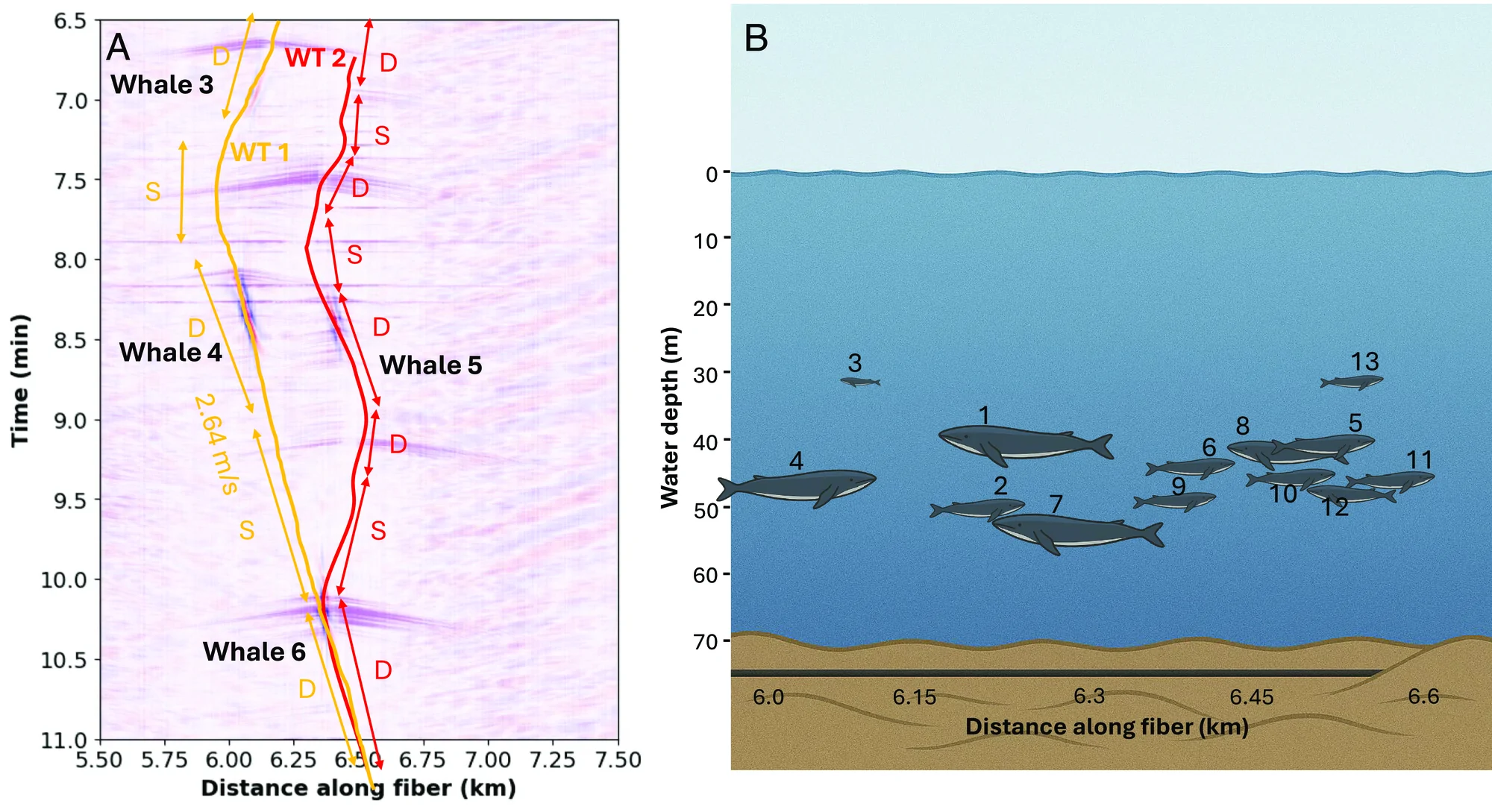
Whale track interpretation and whale location. (*A*) Our interpretation of two whale tracks (WT) over 5.5 min of data. WT1 shows a track containing surface waves and hydrodynamic pressure field signals. WT2 includes vocalizations, surface waves and hydrodynamic pressure fields. Our interpretation of when the whale is diving deep (D) into the water column is indicated (when surface waves and hydrodynamic pressure fields are produced), and when shallow (S) is indicated (whale vocalizes). Note that only the apex of the acoustic signals has been used and that the annotated whales refer to Fig. 5 and Table 1 and are not necessarily four different whales. (*B*) A schematic plot showing the locations of the whales (along the fiber) at their estimated depths as given in Table 1. The sizes are scaled relative to the biggest whale (whale 1). This is an integrated image displaying all whales over the 30 min time window shown in Fig. 5.

Fig. 6 shows a zoomed section (Fig. 6*A*) together with the whales located at their estimated water depths and scaled by their estimated sizes (Fig. 6*B*). Fig. 6*A* illustrates two interpreted WTs, highlighting how whale vocalizations, Scholte wave, and hydrodynamic pressure field signals can be combined to construct plausible movement paths. WT 1 follows the apex of different Scholte waves, whale vocalizations, and the polarization of the maximum amplitude of the hydrodynamic pressure field signals. WT 2 represents an alternative interpretation, using different apex locations and hydrodynamic pressure field signals. These are two of several possible paths for this example. We separate the tracks based on the Scholte waves and the whale vocalizations, as some occur at similar times but at different locations along the fiber. Based on the known blue whale swim speed, it is unlikely that a single whale could generate all recorded signals. In Fig. 6*B*, all 13 identified whale-related signals are placed at their estimated depths and fiber location, and scaled by their estimated sizes. Note that although we display all 13 whales, we do not interpret these as 13 individual whales. As illustrated by the two tracks in Fig. 6*A*, several acoustic, geophysical, and hydrodynamical pressure field signals can be generated by one whale moving along the fiber cable at different water depths. Therefore, the exact number of whales and their paths cannot be uniquely determined from the current data. Further development of the theoretical framework is required to enable more robust tracking of individual animals.

## Discussion

This work investigates DAS signatures generated by the hydrodynamic pressure and velocity fields of moving ships and whales. Ship-generated signals can be independently confirmed using AIS data, whereas the whale-related signals cannot be directly verified without visual observations, tagging, or satellite imagery. Nevertheless, multiple lines of analysis support the interpretation that whales are responsible for the observed signals.

A common approach for identifying whales is to analyze spectrograms and search for stereotypical acoustic signatures. Different whale species produce distinct vocalizations, which can be recognized using spectrograms. In this case, we observe downsweep signals (e.g., from 60 to 20 Hz), which are characteristic of blue whales. Furthermore, these downsweep signals occur just before or just after the hydrodynamic pressure field signals, indicating that both originate from the same moving source.

A second line of evidence is the difference in low-frequency content between whale- and ship-generated signals. The whale-related signals consistently exhibit higher frequencies than those observed for ships (Figs. 4 and 5*D*), which is consistent with the proposed scaling between characteristic length, speed, and observed frequency. Additionally, no ship signatures are present in the time window studied, as confirmed by AIS data. This reduces the likelihood that the observed low-frequency signals originate from vessels.

A third line of evidence is the similarity in *f*–*κ* signatures between whale- and ship-generated signals. In both cases, the dominant energy follows a linear f=Vκ relationship, consistent with a translating hydrodynamic pressure field from a moving source rather than dispersive surface gravity waves.

The estimated depths at which the blue whales generate the low-frequency signals are consistent with reported diving depths for this species (9, 45). At these depths, we do not observe acoustic vocalizations but instead detect slow-moving Scholte waves, which we interpret as being generated by whales moving deeper in the water column, potentially close to the seafloor. The hydrodynamic pressure field signals are also not observed during periods of vocalization. Blue whale vocalizations are typically produced at depths of 20 to 40 m below the sea surface (6, 39), suggesting that the whales must be at greater depths to generate the detectable low-frequency hydrodynamic pressure signals observed in this study.

Finally, we investigate how the signal amplitude varies along the fiber. Both ODEN and LCC produce responses that are wider and have higher amplitudes than those associated with the whale signals. This is consistent with the larger size of the vessels and the expected scaling of the spatial extent of the signal, as reflected by the distance between the zero-crossings adjacent to the maximum amplitude. However, a limiting case arises as the objects move toward the fiber (due to the averaging over the gauge length). In such cases, the distance between zero-crossings adjacent to the maximum amplitudes becomes larger than expected (*SI Appendix*, section S13).

The main limitation of using DAS to detect the hydrodynamic pressure field generated by objects in the sea is its detection range. Kvitungen is the smallest ship investigated in this work and is barely observed above the noise floor for a water depth of ∼75 m. Blue whales are even smaller and are unlikely to be detected at distances greater than approximately 30 to 40 m from the fiber. Larger ships generate stronger signals; however, the signal amplitude decreases with increasing distance from the fiber. By analyzing LCC as it sails at different water depths, we find that the amplitude decays approximately as $1/r^{3}$ (*SI Appendix*, section S14). Using the same data-set, we estimate the detection limit to be approximately 550 m. However, future improvements to the DAS firmware are expected to reduce the 1/f noise increase in strain data. Preliminary results show a one-order-of-magnitude improvement in the 0.01 to 0.1 Hz band, likely to extend the detection range while also detecting more environmental noise sources in the same frequency window.

The current range of the DAS method yields large detection areas for hydrodynamic pressure field signals. If we acquire data over 80 km using one IU and detect LCC 0.55 km from the DAS array, the total area in which the ship can be detected is 44 km^2^. However, new technologies are being developed that can significantly increase the DAS range. Rønnekleiv et al. (32) demonstrate a range of 2,227 km using dedicated DAS repeaters. Mazur et al. (33) show that it is possible to exploit existing repeaters in seabed fiber to perform fiber sensing and achieve ranges exceeding 7,000 km. If this can be demonstrated for the low frequencies observed in this work, it could imply the possibility of tracking whales close to the seabed in remote areas.

## Conclusions

In this study, we observe a unique low-frequency signal recorded using DAS. This signal is interpreted as being caused by hydrodynamic pressure and particle velocity variations created by a ship moving on the sea surface or by a whale swimming close to the seabed fiber-optic cable being interrogated.

To better understand the physics behind the hydrodynamic pressure and particle velocity signature, we model it using Rayleigh’s work on the pressure development during the collapse of a cavity in an incompressible liquid. The response is computed for a single time instance and then translated along the trajectory of the moving source. The final model is obtained by superposing the responses over time. This phenomenological modeling captures the first-order behavior of the observed DAS signals. This includes their kinematics, the *f*–*κ* trends and spatial variation along the fiber. We also use the proposed theory to estimate characteristics such as speed, depth, and length of the objects. We use data from three ships to validate the model. One ship is used for calibration, and the other two for model testing. Finally, the model is applied to data we interpret as originating from silent blue whales.

For ships, we find that the recorded signal is monochromatic and nondispersive, allowing the dominant frequency to be used to determine the ship’s speed, given the known ship length. We test this for the three ships and find that the speed estimates agree with the measured values. However, the speed of one ship is underestimated, likely because it accelerates 1 to 2 min before the interrogator records the signal. For blue whales, we find swimming speeds between 2 and 4 m/s. The observed *f*–*κ* domain signature for the moving hydrodynamic pressure and velocity field is a linear ridge and can be used to estimate apparent velocities. The observed ridges in the domain are slightly smeared but result in speeds consistent with our estimates and AIS-derived values.

The whale’s depths and lengths are estimated using a ship for calibration of the model prior to the estimation. All whales are estimated to swim close to the sea-floor (within 42 m). Using one ship for calibration, we find that the estimated ship lengths and the majority of the whale lengths agree reasonably well with their expected values. However, there are whales estimated to be abnormally long (>30 m) and surprisingly small (<14 m). This underlines that our simple model needs further development to obtain more precise results.

The detection range is found to be proportional to the object’s surface area times its speed. Furthermore, the recorded strain signal decays as one over distance cubed according to our model. This trend is confirmed by observations of a ship sailing at different water depths. For this study, we estimate a detection range of ∼550 m for large ships (150 m long and beam of 28 m) and 42 m for blue whales.

Finally, we find that three detection methods can be utilized for improved whale monitoring: acoustic pressure signals generated by whale vocalization can be used to track whales when they are close to the sea surface, whereas when the whale is closer to the seabed, Scholte waves and hydrodynamic pressure and velocity field signals can be used. This complementarity opens exciting ways to track whales in the future.

## Materials and Methods

The DAS data are analyzed using various methods, with key theoretical elements described in this section. First, the data processing is presented. Second, the modeling of fiber strain induced by a hydrodynamic pressure field from a moving source within or on top of the water column is presented. Finally, the description of the uncertainty analysis of the estimated values is given.

### Data Processing.

The data are preprocessed using the following steps. First, the data are converted from raw time-differentiated phase-change data to strain (46). Second, the strain data are resampled to 100 Hz for the ships and 156 Hz for the whales, then a zero-phase order-2 Butterworth band-pass filter is applied to focus on the various signals in the data. For the low-frequency signals different cut-off frequencies were used depending on the application. However, all frequencies were between 0.007 and 0.22 Hz. For higher frequency signals within 1 Hz and 90% of the Nyquist frequency are used. Tukey tapering is used before the filtering. Finally, the interrogator’s common-mode noise was reduced by computing a model for all time samples using the median value between 30 and 50 km. The model is then subtracted from the DAS data.

### Modeling of Strain from a Moving Hydrodynamic Particle Velocity Field.

To better understand the low-frequency data observed for moving whales and ships, we model the response using a simplified radial flow formulation based on Rayleigh’s work on pressure development during a cavity collapse in an incompressible liquid (35). We adopt Rayleigh’s formulation as a simplified representation of how the velocity field decays with distance from a localized disturbance generated by a moving object. To represent this moving object, we construct the total response as a superposition of local velocity fields along the object’s trajectory. This approach provides a first-order model of a hydrodynamic pressure field that travels with the source and interacts with the seabed and the fiber. *SI Appendix*, Fig. S20 shows a schematic illustration of the problem at a single time instance, along with the essential parameters. We derive and present equations for this single instance example.

Let *V* and *R* be the velocity and radius, respectively, of the inner boundary of the object at time *t*, and *v* be the particle velocity at a distance *r* from the center of the cavity (or bubble). Under the assumption of incompressible radial flow, we obtain:

[1] $$\begin{array}{c} \frac{\upsilon }{V}=\frac{R^{2}}{r^{2}}. \end{array}$$

We assume that the in-line component coincides with the *x*–axis and hence need to model the $v_{x}$ component of the particle velocity. From *v* we can compute $v_{x}$ by projection:

[2] $$\begin{array}{c} \upsilon_{x}=\upsilon \;\text{cos}\;\theta . \end{array}$$

The final expression is obtained by setting cosθ=x/r and inserting Eq. **1** into Eq. **2**:

[3] $$\begin{array}{c} \upsilon_{x}=V\;R^{2}\frac{x}{r^{3}}. \end{array}$$

To account for temporal variability of the source, we introduce a source function S(t,x), which represents the time-dependent forcing associated with a moving object:

[4] $$\begin{array}{c} \upsilon_{x}=V\;R^{2}\frac{x}{r^{3}}S\left(t,x\right). \end{array}$$

The source function can, for example, be a Ricker wavelet or a sinusoidal function. A sinusoidal function is used in this work. Moreover, to account for the motion of the source, the source function can be represented as a translating field:

[5] $$\begin{array}{c} S(t,x)=S(x-V\;t). \end{array}$$

This expression assumes that the dominant structure of the hydrodynamic velocity field moves with the source and remains unchanged. To construct the total response by superposing all time instances along a ship trajectory, we introduce:

[6] $$\begin{array}{c} \upsilon_{x}\left(t,x\right)=\sum_{n}V_{n}R_{n}^{2}\frac{x-x_{n}}{{\left[{\left(x-x_{n}\right)}^{2}+{\left(y-y_{n}\right)}^{2}+{\left(z-z_{n}\right)}^{2}\right]}^{3/2}}S\left(t-t_{n}\right), \end{array}$$

where $\left(x_{n},y_{n},z_{n}\right)$ is the source position at time step *n*, and (x,y,z) are the coordinates of the fiber channel. In Eq. **6**, the source term is the discrete representation of Eq. **5**.

DAS records the in-line strain component. Therefore, we relate the particle velocity for one time instance (for simplicity) in Eq. **4** to strain. The axial strain is defined as

[7] $$\begin{array}{c} \epsilon_{x}=\frac{d}{\mathrm{dx}}u_{x}, \end{array}$$

where $u_{x}$ is the particle displacement. The displacement is related to the particle velocity through time integration:

[8] $$\begin{array}{c} u_{x}=\int \upsilon_{x}dt. \end{array}$$

We can then insert Eq. **8** into Eq. **7** to get an expression linking strain to particle velocity.

To better understand how the signal changes when a whale, or a ship, is directly above the fiber or far away from the source, we derive the analytic strain-rate expression by differentiating the particle velocity in Eq. **4** with respect to *x* (assuming S(t,x)=1). To simplify the analysis, we consider strain-rate, which is directly obtained from the spatial derivative of the particle velocity:

[9] $${\dot{\epsilon }}_{x}=\frac{d\upsilon_{x}}{dx},$$

[10] $${\dot{\epsilon }}_{x}=V\;R^{2}\frac{y^{2}+z^{2}-2x^{2}}{{\left(x^{2}+y^{2}+z^{2}\right)}^{5/2}}.$$

If we now consider the case when the object is directly above the source (x=y=0) we get

[11] $$\begin{array}{c} {\dot{\epsilon }}_{x}=\frac{V\;R^{2}}{z^{3}}, \end{array}$$

which shows that the strain-rate amplitude decreases as the cube of the water depth. A second case to be considered, is when the signal is recorded at a distance along the fiber from the source, i.e., x≫z and y=0. The results in the following expression:

[12] $$\begin{array}{c} {\dot{\epsilon }}_{x}=\frac{-2V\;R^{2}}{x^{3}} \end{array}$$

indicating both a decay proportional to $x^{-3}$ and a sign reversal. Eq. **10** is visualized in *SI Appendix*, Fig. S19.

We can further use Eq. **10** to estimate the characteristic length of whales and ships. This is done by computing the zero-crossing by setting Eq. **10** equal to zero:

[13] $$\begin{array}{c} x=\sqrt{\frac{y^{2}+z^{2}}{2}}, \end{array}$$

where *y* is the out-of-plane horizontal spatial coordinate (perpendicular to *x*), or the cross-line distance from cable to source. If we assume that the object moves directly above the fiber (y=0), we have

[14] $$\begin{array}{c} x=\frac{z}{\sqrt{2}}. \end{array}$$

When the object crosses the fiber, the strain-rate amplitude is given by Eq. **11** and using Eq. **14** allows us to derive an expression for the characteristic length of the object:

[15] $$\begin{array}{c} L={\left(\frac{{\dot{\epsilon }}_{x}x_{b}^{3}}{8\sqrt{2}f\alpha }\right)}^{1/3}, \end{array}$$

where the following expressions have been used: $x_{b}=2x$ is the spatial full width between the two zero-crossings adjacent to the maximum value. We further approximate the effective cross-sectional area of the object as $R^{2}=2LW$ for whales and $R^{2}=LW$ for ships, where *W* is the object width and α=W/L the aspect ratio (*SI Appendix*, Fig. S17). Finally, we relate the object speed to its characteristic length by V=2Lf, assuming that the observed signal frequency reflects the passage of the object above the fiber (Eq. **21**). This formulation provides a first-order estimate of the ship or whale length based on DAS observations alone.

Eq. **15** is a special case where whales and ships cross directly above the fiber. If this is not the case, the out-of-plane horizontal spatial coordinate *y* is nonzero and can be computed by rearranging Eq. **13** and using that $x=x_{b}/2$:

[16] $$\begin{array}{c} y=\sqrt{\frac{x_{b}^{2}}{2}-z^{2}}. \end{array}$$

For the special case when y=0, this relation reduces to $x_{b}=\sqrt{2}z$. However, our empirical data show that this scaling does not adequately describe the observations, especially for large water depths (z>100 m; see *SI Appendix*, Fig. S16*B*). For such water depths, we introduce an empirical scaling relation:

[17] $$\begin{array}{c} x_{b}=\chi z, \end{array}$$

where the theoretical value for *χ* is $\sqrt{2}$, while our measurements show a lower value of χ≈0.66. To account for this discrepancy, we replace $\sqrt{2}$ with the empirical factor *χ*, which changes Eq. **17** to:

[18] $$\begin{array}{c} y=\sqrt{{\left(\frac{x_{b}}{\chi }\right)}^{2}-z^{2}}. \end{array}$$

The discrepancy between the model and the measured data likely reflects the simplifying assumptions made in the current formulation. In particular, the assumption of radial symmetry may not adequately capture the complex hydrodynamic response of ships and swimming whales. The smaller *χ* value suggests that the effective spatial extent of the hydrodynamic disturbance is narrower than predicted by the spherical model. A more realistic description may require including directional (nonspherical) effects in the hydrodynamic pressure field, which we leave for future work.

If a signal with unknown depth *z* is recorded with amplitude *A*, and another signal with known depth (e.g., a ship; $z_{s}$) where the amplitude ($A_{s}$) is available, we can compare the two and estimate the unknown depth. We assume that each object passes directly above a channel in the DAS array. Using the amplitude scaling in Eq. **11**, we obtain

[19] $$\begin{array}{c} \frac{A_{s}}{A}=\frac{V_{s}R_{s}^{2}}{z_{s}^{3}}{\left(2\frac{VR^{2}}{z^{3}}\right)}^{-1}. \end{array}$$

By isolating the unknown *z* we find:

[20] $$\begin{array}{c} z={\left(2V\;R^{2}\frac{z_{s}^{3}}{V_{s}R_{s}^{2}}\frac{A}{A_{s}}\right)}^{1/3}. \end{array}$$

This approach provides a practical method for estimating source depth using relative amplitude measurements. Eq. **20** is most applicable to the case of the fully submerged whales, where the hydrodynamic field can be approximated as symmetric around the source. For ships, which are surface-bound, this symmetry is not present, and the effective scaling may differ by a constant factor. In this work, this difference is approximated by a factor of 1/2, reflecting the reduced symmetry of the flow field. The sizes of the reference object (ship) and the unknown object (blue whale) are assumed to be known. Such a scenario occurs when investigating a vessel with available AIS data or when whales are identified through acoustic recordings, spectrograms, or visual observations. Moreover, the speed of a ship can be estimated in two ways. First, it can be estimated directly from AIS data. Second, if the AIS data are unavailable, it can be estimated by using the relation between speed and the characteristic frequency. Assuming that the ship generated a disturbance over its length *L*, followed by a return flow of similar extent, the dominant frequency is given as

[21] $$\begin{array}{c} f=\frac{V}{2L}. \end{array}$$

A more complete description would require a comprehensive fluid-flow analysis of a ship moving at a given velocity, which is beyond the scope of this work. Here, we employ a first-order model to capture the leading-order effects of fluid motion while retaining the key physical mechanisms that explain the observed DAS signatures. A natural extension of this framework would be to replace the constant velocity assumption with a velocity function that varies with time. This would, for instance, allow the inclusion of a contraction phase after the ship or whale has passed the fiber, analogous to the models developed for expanding air bubbles generated by seismic air guns, where the contraction phase of the bubble is also accounted for.

### Uncertainty Analysis.

To quantify uncertainties in water depth estimates, errors in the calculated velocities are propagated using Monte Carlo sampling. Frequency uncertainty is represented via the SD of the maximum value of each channel containing the low frequency signal, whereas the uncertainty in the whale size is chosen such that it varies within known values (25 to 27 m). Moreover, the uncertainty in the maximum amplitudes is estimated by first extracting the top 1% of amplitudes and then computing the mean and SD. The speed of the blue whales and the ships is then computed by drawing 1,000 independent samples of object length and frequency, using Eq. **21**. The water depth is computed using the same procedure, drawing 1,000 independent samples of the amplitude and speed, and using Eq. **20**. The length is similarly computed using Eq. **15**.

### Use of AI.

ChatGPT (OpenAI, GPT-5.3) was used to assist with text editing and to improve clarity and flow of the manuscript. All AI generated edits were reviewed and validated by the authors. AI tools were not used to generate scientific content, interpret results, or draw conclusions.

## Supplementary Material

Appendix 01 (PDF)

## Data Availability

A resampled version of the dataset is available in the DataverseNO repository (https://doi.org/10.18710/H8MNKT). The code used to obtain the results presented is shared in the same repository. The AIS data used will also be shared, but is openly available at Kystdatahuset.no (47).
