# Supplementary material for: Detecting silent whales using seabed fiber-optic cables
Source: Proc Natl Acad Sci U S A. 2026 Jun 23;123(26):e2603077123. doi: 10.1073/pnas.2603077123 (PMC13321103; doi:10.1073/pnas.2603077123)
Supplement: Supplementary file 1 — Appendix 01 (PDF) [file pnas.2603077123.sapp.pdf]

Supporting Information for

Detecting silent whales using seabed fibre optic cables

Robin André Rørstadbotnen and Martin Landrø

Robin André Rørstadbotnen .
E-mail: robin.a.rorstadbotnen@ntnu.no

This PDF file includes:

Supporting text
Figs. S1 to S20
Table S1
SI References

Supporting Information Text

S1. Introduction to the Supplementary Material

This document contains supplementary information meant to support the ideas, results, discussion, and conclusion of the work presented in the main text. First, section S2 dives into the difference between Kelvin wakes and a moving pressure field generated by a sailing vessel or a swimming whale. Section S3 presents the time-distance representation of the ship's acoustic signatures as recorded by distributed acoustic sensing (DAS). Section S4 presents acoustic and hydrodynamic pressure field data from unburied and buried cable segments. Section S5 shows and discusses the f–κ representation of the modelling given in Fig. 3 of the main text. Section S6 shows the frequency-wavenumber representation of the t–x data in section S3. Section S7 presents a theoretical description of how ocean surface waves decay with depth. Section S8 shows the power-spectral-density of the ship and whale examples and how it can be used to estimate their sailing and swimming speeds. Section S9 show spectrograms for different frequency bands, indicating which baleen whale is generating the various observed signatures. Section S10 presents the AIS tracks during the whale vocalisation, showing that none of the tracks overlaps with the observed signatures. Section S11 shows combined plots where high and low-frequency representations are overlaid. These are used to understand the relationships between the recorded signatures. In Section S12, amplitude-along-fibre responses (or amplitude-versus-offset) are presented for both ship and whale examples. Section S13 discusses the limiting case when the vertical distance from the closest DAS channel to the submerged object goes towards the gauge length value. Section S14 shows how the amplitude decays as a function of distance extracted from the data. Section S15 presents the strain rate model along the $x-y$ plane for a given water depth. Section S16 presents the schematic used to describe the proposed fluid dynamical model. Finally, Section S17 presents the aspect ratio for different whale species and an estimation of their detection range based on the estimated blue whale detection range found in this work.

S2. Kelvin wake or a moving hydrodynamic pressure field?

A point pressure source moving steadily over a calm, deep water surface generates a characteristic wake pattern confined to a 39° wedge. This pattern is normally called the Kelvin wake (1). The Kelvin wake describes a wave system that is stationary in a ship-fixed reference frame and consists of two main components: (1) a traverse wave propagating approximately in the direction of the ship; (2) divergent waves propagating at an angle relative to the ship track. The typical frequency range reported for Kelvin wake signals ranges from 0.1 to 0.5 Hz (2, 3). Once detached from the ship hull, however, these waves propagate freely as surface gravity waves at a phase velocity determined by the local water depth (e.g., under deep water conditions, $v_p = \sqrt{gh}$), which can exceed the ship speed and result in lower observed frequencies. In contrast, the wave frequencies observed in the present study are lower than both the frequencies typically associated with Kelvin wakes and those predicted by free surface gravity wave propagation at the local water depth.

The Froude number is the ratio between the speed of a moving object and the characteristic gravity-wave speed determined by the relevant length scale. In ship hydrodynamics, two commonly used Froude numbers are the length-based and the depth-based Froude numbers. The length-based Froude number is given as:

$$F_l = \frac{V}{\sqrt{gL}} \qquad [1]$$

where L is the length of the ship (same order as the wavelength, λ), g is the gravitational acceleration, and V is the sailing speed of the ship. This parameter is typically used to characterise wave-making resistance and the classical Kelvin wake regime (3).

However, the depth-based Froude number indicates whether depth effects are important. The depth-based Froude number is given as:

$$F_h = \frac{V}{\sqrt{gh}} \qquad [2]$$

where h is the water depth (3, 4). The ships analysed in this work sail at speeds from 4.5 m/s to 7.0 m/s, but at similar water depths (70–80 m). Plugging these numbers into Eq. (2) results in the following depth-based Froude numbers: $F_{\mathrm{HH}} = F_{\mathrm{ODEN}} = 0.16$, $F_{\mathrm{LCC}} = 0.25$. As $F_h \ll 1$ in all three cases, the vessel does not operate in the shallow-water regime ($F_h \sim 1$), and it is therefore unlikely that shallow-water hydraulic effects should occur.

Next, we investigate whether the observed waves satisfy the deep-water condition. Kelvin wakes consist of surface gravity waves, and their dispersion can be described by the linear surface gravity-wave relation (5). For a steadily moving pressure disturbance in deep water, the dominant transverse wavelength is given by (6):

$$\lambda \sim \frac{2\pi V^2}{g}. \qquad [3]$$

As mentioned, the ship's sail speeds here range from 4.5 to 7.0 m/s, corresponding to wavelengths of 13.0 to 31.4 m. The dispersion relation for linear surface gravity waves is given by (5, 7):

$$\omega^2 = gk \tanh{(kh)} \qquad [4]$$

where ω is the angular frequency, k the angular wavenumber and h the water depth. Deep-water conditions are satisfied when $kh \gg 1$, which means $\tanh(kh) \sim 1$ and Eq. (4) becomes:

$$\omega^2 = gk. \tag{5}$$

For the water depth in this work, 70–80 m, we find:

$$kh = \frac{2\pi h}{\lambda} \sim 15 - 35 \tag{6}$$

and obtain $\tanh(kh) \sim 1$. The wave field can be considered to be in deep water.

Based on the above, we can continue our analysis under deep-water conditions. In deep water, the classical Kelvin wake theory applies (3, 8). Moreover, we use this theory to estimate the expected frequency from the ship sailing speed, and vice versa. We start by introducing the phase velocity (v_p) relation for a steadily moving pressure disturbance (e.g., a ship) (3):

$$v_p = \frac{\omega}{k} = V\cos\theta \tag{7}$$

where θ is the angle of propagation relative to the sailing line, and V is the sailing speed. To obtain an expression that relates frequency to sail speed, we use the deep-water approximation and the dispersion relation reduces to Eq. (5). Combining this with Eq. (7) we find:

$$\omega = \frac{g}{V\cos\theta}. \tag{8}$$

This results in a minimum frequency of 0.22–0.34 Hz for the ships studied in this work. However, the frequencies observed in this study (∼0.02 Hz) are more than an order of magnitude lower than the minimum Kelvin wake frequencies we expect here. We can now re-arrange Eq. (8) to estimate ship velocity. From the observed frequencies recorded during the passage of ODEN and LCC, the vessels would need to sail at speeds of 65.3 and 73.0 m/s, respectively, to explain the measured values under Kelvin wake theory. These are unrealistically high speeds. Once detached from the ship hull, however, the generated surface gravity waves propagate independently at a speed determined by the local water depth, given by $v_p = \sqrt{gh}$. For a water depth of 76 m, this yields a phase velocity of approximately 27 m/s, exceeding the speeds of both vessels. The corresponding minimum frequency is 0.058 Hz, which is considerably lower than the Kelvin wake estimates based on ship speed, yet still nearly three times higher than the frequencies observed here. Furthermore, a lower frequency signature is expected at water depths down to 400 m, where the phase velocity would be 62.6 m/s, yielding a minimum frequency of 0.025 Hz. This frequency is close to our observations. However, since the observed frequency remains constant across all water depths, it cannot be tied to a depth-dependent phase speed. Fig. S1 shows the implementation of Eq. (8) across a range of frequencies and angles, with the relevant speeds and frequencies under Kelvin wake theory highlighted. Taken together, the observed low-frequency signatures in the DAS data cannot be attributed to classical dispersive Kelvin wake waves or a surface gravity wave origin. Instead, we interpret these observations as consistent with a moving hydrodynamic pressure field.

A moving hydrodynamic pressure field can be generated by objects travelling through or on top of the ocean, such as vessels or whales. Such a field does not necessarily manifest as a dispersive surface gravity wave. A spatial pressure field that propagates past a fixed DAS virtual sensor at speed V produces the following frequency–wavenumber relation (9)

$$f = V\kappa, \tag{9}$$

where f is frequency, κ is the spatial wavenumber expressed in cycles per metre, and V is the speed of the travelling object. The relation in Eq. (9) differs from the dispersive gravity-wave relation in Eq. (4), as it represents the kinematic signature of a translating pressure field rather than a propagating surface wave. In the frequency–wavenumber domain, dispersive gravity waves follow a curved dispersion relation, whereas the moving pressure field from a ship passing appears as a linear line with slope equal to the travelling speed. This is clearly observed in Fig. 3 in the main text, where the ocean surface gravity-wave signature is curved, while the kinematic ship signature is linear.

In addition to the unrealistic ship speeds estimated from Eq. (8), the observed low-frequency strain signals do not follow the dispersive gravity-wave relation. For a deep-water Kelvin wake, the frequency–wavenumber relation must satisfy both the deep-water dispersion relation (Eq. (5)) and the stationary phase condition (Eq. (7)). Together, these relations produce a curved dispersion structure in frequency–wavenumber space. In contrast, the low-frequency strain energy shown in Fig. S2 follows the linear relationship given by Eq. (9). This linear relationship is characteristic of a spatial hydrodynamic pressure field propagating past a fixed sensor (in our case, a virtual DAS channel). The slope of the linear ridge corresponds to the sailing speed of the vessel generating the disturbance. We therefore interpret the low-frequency signatures as coming from a moving hydrodynamic pressure field rather than from Kelvin wake waves.

Fig. S2b shows the frequency–wavenumber plot generated by a whale (whale 6 in Fig. 5, main text). Similar to the case for LCC in Fig. S2a, the ocean waves are dispersive and follow a curved path, whereas the non-acoustic moving hydrodynamic pressure field produces a linear trend. If the low-frequency energy were generated by dispersive ocean surface gravity waves (e.g., Kelvin wakes or wind waves), the energy distribution in the frequency–wavenumber domain should be curved. Instead, the observed whale-associated energy closely resembles the ship signal, but with higher temporal and spatial frequency content.

The slope of the linear line corresponds to speeds of approximately 2.5 to 4.5 m/s, consistent with reported swimming speeds of blue whales and lower than those from the vessels. We therefore interpret the linear 0.05–0.08 Hz signature as arising

from a moving hydrodynamic pressure field generated by the swimming whale. As the whale moves through the water column, this pressure field moves with it and, when passing the fibre, produces a kinematic frequency–wavenumber signature similar to that of the ship: $f = V\kappa$, where V is the swimming speed of the whale. The observed behaviour is thus consistent with a translating hydrodynamic pressure field rather than dispersive surface gravity waves.

S3. Acoustic signatures for ships

Fig. S3 shows the acoustic signature of four ships of different sizes, observed at roughly the same water depth (70–80 m) and sailing speed. Note that all displays have been band-pass filtered between 1 and 45 Hz. It is eminently clear that the observed amplitude scales with the size of the ship. The smallest ship is Kvitungen (45 m long, 8 m beam wide), and the amplitude of this ship is barely above the noise floor. Helmer Hansen is 2.3 times bigger and generates a stronger acoustic signal. The icebreaker ODEN is 4.0 times bigger than HH, also evident through stronger strain levels. The only exception is that the largest ship, Le Commandant Charcot, produces acoustic amplitudes similar to ODEN. Note that the distance from the ships (based on the AIS track) to the closest virtual DAS channel is indicated by grey filled circles, and the AIS track relative to the fibre cable position is given in the insets (see Fig. 1 of the main text for the location of the inset).

S4. LCC recorded on buried and unburied fibre segments

The 2022 DAS test at Svalbard yielded data across different marine environments, providing valuable insights into the detectability of the low-frequency hydrodynamic pressure signals. In this section, we analyse data recorded from Ny-Ålesund on the inner and outer fibres (see Fig. 1 in the main text). These fibres are both unburied (west of Prins Karls Forland) and buried (in Kongsfjorden). Fig. S4 shows data from segments where both the inner and outer fibres are unburied. Using the AIS of LCC, we first identified where the ship crosses the inner fibre, then the outer fibre. Both crossings are detectable acoustically and for the hydrodynamic pressure signal. The main difference from the buried fibre segments is the lower detection range in both frequency bands. There is a clear difference between Fig. S4c and d. In Fig. S4c, the signal is coherent for a 100–200 m, whereas Fig. S4d shows the pressure signal but with less coherence. We interpret this as variation in coupling: Fig. S4c is recorded on a partly buried segment, whereas Fig. S4d is completely unburied. This differs from the reported unburied fibre for this part of the fibre routes. The same pattern can be observed for the background ocean waves in Fig. S4c, which are coherent over some fibre segments (e.g., from 62.1–62.2 km) and incoherent over other segments (e.g., from 62.2–62.3). In Fig. S4d, the swell is hardly observable.

To confirm the observations of LCC in Isfjorden (Fig. S3 and Fig. 2 of the main text), we investigate when the vessel sails close to the inner fibre in Kongsfjorden. Fig. S5 shows the associated data (for water depths around 300 m) where LCC is detectable for a 7 km section of the fibre. The acoustic and low-frequency signatures are clearly observed, with amplitude varying with the ship's cross-line distance (transparent circles overlaid on the DAS data). This data also indicates LCC's detection limit. Between 21 and 22 km the vessel is ∼550 m from the fibre, and the signal is slightly above the noise floor, i.e., the maximum distance we can detect LCC is around 550 m.

The data recorded on the unburied segment confirm that the fibre does not need to be buried to record low-frequency hydrodynamic pressure fields. The data recorded in Kongsfjorden show that low-frequency signals can be detected over a substantial distance and are indeed observable on two independent DAS systems recording 2 years apart.

We analyse this 7 km segment further by extracting the spatial full width for each time sample. Fig. S6a shows the resulting picks, where green dots indicate the maximum energy and the black dots show the adjacent zero-crossings (Fig. S7 shows two examples of how the zero-crossings are picked). Note that data between 800 and 950 s had too low signal-to-noise ratios to reliably detect the zero-crossing and were excluded from the analysis. The spatial full width can be used to compute the cross-line distance from the fibre to the vessel using Eq. (18) in the main text. Fig. S6b depicts the resulting cross-line distance and compares it to the distance computed from the AIS data. The overall trend estimated from the low-frequency DAS data agrees reasonably well with that of the AIS data. However, data points are discarded because they yield imaginary values, highlighting the need for further work on this theory to improve estimates and make them more robust.

Fig. S7 show traces containing the maximum value for LCC crossing the fibre in two different locations. The trace in Fig. S7a is a crossing where the water depth is 82 m, whereas the crossing in Fig. S7b is for 413 m water depth. For the shallow-water crossing, the raw trace shows a clear maximum value, and the adjacent zero crossings are evident. The low-frequency signal recorded in deeper waters is more attenuated by geometrical spreading and is of lower signal-to-noise ratio. The maximum value and zero-crossings are hard to reliably pick from the raw trace, so a 5-sample moving mean is applied to extract them. Therefore, shallow water crossings yield a more reliable x_b value than deeper water crossings. Note that each zero-crossing is obtained by linear interpolation using the adjacent positive and negative amplitude channels.

S5. Frequency-wavenumber representation of modelling results

Fig. S8 shows the f–κ representations of the modelling given in Fig. 3 of the main text. These demonstrate that the apparent f–κ signature depends strongly on the angle between the vessel trajectory and the fibre. When the vessel is sailing nearly parallel to the fibre, the energy collapses onto a line consistent with $f = V\kappa$, allowing reliable estimation of the sailing speed used in the modelling. In contrast, for larger crossing angles, the response becomes increasingly smeared in the f–κ domain. Extracting the sailing speed is most challenging for perpendicular crossings.

S6. Ship signatures in the frequency-wavenumber domain

Fig. S9 shows frequency-wavenumber plots covering 0 and 50 Hz and $\pm 0.12\ \text{m}^{-1}$ in horizontal wavenumbers. Fig. S9a depicts the background noise conditions, where only ocean surface gravity waves (OSGW) (10) and resonances within the sedimentary layer (11) directly under the fibre are present. Fig. S9b, c, d show signatures of three ships of increasing size. Each ship produces unique frequency components, with some dominating specific frequency bands. Kvitungen has components every 5 Hz, while LCC has components every 12.4 Hz and at 39.8, 40.7, and 44 Hz. ODEN shows the greatest diversity in frequency components, which are slightly more smeared, and also shows elevated energy following the sound speed in water (indicated by the yellow line in Fig. S9). The same trend as in Fig. S3 is observed, i.e., that ODEN and Le Commandant Charcot generate the strongest amplitudes and Kvitungen the smallest.

S7. The expected decay of ocean surface wave energy with depth

The horizontal particle velocity is obtained from linear Airy wave theory by differentiating the velocity potential (see, e.g., Mei et al. (12)):

$$\Phi = \frac{gA}{\omega}\frac{\cosh(k(z+h))}{\cosh(kh)}e^{i(kx-\omega t)}. \qquad [10]$$

Differentiating with respect to x, we obtain:

$$v_x = \frac{\partial \Phi}{\partial x} = \frac{igkA}{\omega}\frac{\cosh(k(z+h))}{\cosh(kh)}e^{i(kx-\omega t)}. \qquad [11]$$

Here, g is the gravitational acceleration, A is the wave amplitude, $\omega = 2\pi f$ is the angular frequency, $k = 2\pi\kappa$ is the angular wavenumber, and z is the vertical coordinate. To obtain an expression for strain, we first differentiate v_x with respect to x to obtain the strain rate:

$$\dot{\epsilon}_x = \frac{\partial v_x}{\partial x} = -\frac{k^2 gA}{\omega}\frac{\cosh(k(z+h))}{\cosh(kh)}e^{i(kx-\omega t)}. \qquad [12]$$

We then carry out a time integration to find an expression for strain:

$$\epsilon_x = \frac{ik^2 gA}{\omega^2}\frac{\cosh(k(z+h))}{\cosh(kh)}e^{i(kx-\omega t)}. \qquad [13]$$

Since we are only interested in the amplitude variation with depth, we consider the magnitude of Eq. (13), $|\epsilon_x|$. We evaluate this magnitude for varying water depth h and compare it to the trend obtained in this work. Fig. S10 shows the expected decay of strain amplitude with water depth for ocean surface gravity waves of different wavelengths, compared to the observed $1/h^3$ trend. Short wavelengths representative of Kelvin wakes ($\lambda = 13$ and 31 m) exhibit rapid attenuation with depth, becoming negligible within the upper ∼100 m. In contrast, a longer wavelength ($\lambda = 100$ m) can be observed deeper in the water column. However, it still decays faster than what the observed trend would suggest. The $1/h^3$ curve shows a much slower decay, consistent with the behaviour of the low-frequency signals observed in this study.

Rørstadbotnen and Landrø (10) observed OSGW waves in Kongsfjorden and offshore Forlandet. In Kongsfjorden, the fibre is located at water depths between 250 and 300 m, and wavelengths ranging from 200 to 1000 m (wavenumbers from 0.001 to 0.005 m^{-1}) are observed during periods of strong winds. Offshore Forlandet, where the fibre is deployed in shallower water (70–80 m), shorter wavelengths from 80 to 1000 m (wavenumbers from 0.001 to 0.012 m^{-1}) are observed during strong wind-sea conditions. Furthermore, Landrø et al. (13) present strain signals recorded at different water depths for several distant storms (see their Fig. 5). These results show that higher frequencies (0.07–0.1 Hz) are observed only at shallower water depths. In contrast, deeper water (>300 m) is dominated by lower frequencies (longer wavelengths). This observation is consistent with Eq. (13) in the main text and Fig. S10.

S8. Object speed from power spectrum density

If we assume that the ship is first pushing the water for its entire length, followed by an opposite back-flow lasting for the same length L, the dominant frequency for one sequence can be expressed as $f = V/2L$, where V is the speed of the ship. By taking the fast Fourier transform (FFT) of time windows that include the low-frequency ship signal, we can extract the amplitude spectrum and the dominant ship frequency to estimate the ship's speed. In Fig. S11b, c, d, this is done for HH, ODEN and LCC and their respective speeds are estimated to 2.9 ± 0.5 m/s, 4.6 ± 0.8 m/s, and 7.2 ± 0.9 m/s.

The same is done for the 13 whale examples introduced in Fig. 5b of the main text. The resulting amplitude spectra are given in Fig. S11a. All 13 whale signals exhibit higher-frequency content than the ship, with frequencies ranging from 0.046 to 0.101 Hz. The resulting swimming speed estimates range from 2.4 to 3.8 m/s, with one outlier at 5.1 m/s. All estimates are given in Table 1 in the main text. Note also the response for whales 10, 11 and 12, where an interference pattern is apparent in the combined spectrum. We speculate that this is due to slightly different frequency content among the different whales, which interfere to create the observed curve. Modelling this pattern is outside the scope of this work, but should be investigated in future research.

S9. Spectrograms of whale vocalisation, Scholte waves and hydrodynamic pressure signals

On 2022.08.24, several whale calls were observed together with Scholte waves and hydrodynamic pressure signals. By investigating the spectrograms given in Fig. S12a, we interpret the calls to be downsweeps from blue whales. The figure shows a range of downsweep signatures, which vary in frequency bandwidth. Most sweeps start at around 60 Hz and end at 20 Hz, which is typical for blue whale calls (14). Fig. S12b shows the low-frequency content of the same time series, and we observe two clear low-frequency events occurring before and after a set of whale vocalisations. Fig. S12c and d focus on a channel containing Scholte wave signatures and the associated low-frequency responses. The first Scholte wave coincides with a low-frequency event. In contrast, the other Scholte waves are not connected to any low-frequency events. No whale calls are observed on this channel.

Note that only three small ships were close to the fibre during this period (according to the AIS database). None of these ships overlaps with the recorded signals. They are at distances exceeding the detection range of LCC and hence expected not to be observed (see section S10 for more information).

S10. AIS tracks during whale vocalisation

During the 35 minutes of whale activity on 2022.08.24, three ships broadcast signals to the AIS (15). The distance and timing of these ships are given in Fig. S13. Fig. S13a shows the AIS track from AEGIR (length and beam width not given but assumed similar to BARBATUS), Fig. S13b for BARBATUS (length 13 m, beam 4 m) and Fig. S13c for KAPS (length 9 m, beam 3 m). None of the AIS tracks overlaps the whale calls, Scholte waves, or the hydrodynamic pressure signals. It is worth noting that no anchoring is allowed in the Adventfjorden area, where the Scholte waves are recorded. The three ships are significantly smaller than Le Commandant Charcot (length 150 m, beam 28 m) and outside its detection range. We investigate both frequency bands in detail for the three ships and confirm that the DAS interrogator picks up neither.

S11. Combining high and low-frequency signatures

Fig. S14 overlays a transparent version of the acoustic signals on top of the low-frequency signals. The figure is divided into three parts; Fig. S14a emphasises the arrivals of acoustic and Scholte waves, while Fig. S14b, c emphasise Scholte waves and hydrodynamic signals. In Fig. S14a, 0.9 min of data focuses on clear blue whale vocalisations (horizontal lines, zoom in inset) that seem to change into Scholte waves at 2.35 min. For this example, a disconnected hydrodynamic pressure signature is observed from 1.8 to 2.1 min (channel at 6.0–6.1 km). We interpret this as a separate non-vocalising whale closer to the sea floor. There is a clear difference in move-out between vocalisations (1440 to 3300 m/s) and Scholte waves (70 to 500 m/s), and little move-out for the hydrodynamic pressure signal, except for the coherent arrival over several channels and time. Fig. S14b and c show two cases of Scholte waves that perfectly overlay the hydrodynamic pressure signal. The reason we observe Scholte waves alongside hydrodynamic pressure at some positions but not others is not fully understood. One possibility is that more Scholte waves are generated where the bathymetry changes rapidly; for example, a seamount can act as a diffractor, producing additional seismic energy. Another possibility is that the whale accelerates and displaces more water. A third possibility concerns the gauge length and its effect on the hydrodynamic pressure signal (see section S13). One fact is clear: the whale needs to be close to the sea floor to produce any observable hydrodynamic pressure fields. Fig. S14a is more complex to interpret, but might be a combination of the causes listed above, in addition to other physical phenomena.

S12. Amplitude along fibre response of ships and whales

The amplitude variation observed along the fibre depends on the distance from the object to the fibre cable. When a vessel or a whale is directly above the fibre, i.e., crossing it, the distance is equal to the water depth at the point for the vessel and the vertical distance from the whale to the closest point on the fibre, i.e., $x = y = 0$ (more info in section S15). Moreover, according to Eq. (10) in the main text, the ship should have a wider response than the whale. We use this equation to model an amplitude variation along the fibre (AVF) response to confirm whether our model fits our observations. Fig. S15 shows the modelled and observed AVF for LCC (Fig. S15a) and a representative whale example, whale 7 (Fig. S15b). Note that the same whale is used for both examples, is not recorded at the same time as LCC (plotted together) and that the modelling is normalised and scaled relative to the maximum amplitude of the data it is compared to. It is evident from the plots that our model agrees well with the observed data, i.e., that a ship on the sea surface has a wider distribution than a whale at 40–60 m water depth.

Fig. S16 focuses the analysis on how the AVF response changes as LCC sails at different water depths. As stated above, according to Eq. (10) in the main text, a larger water depth should result in a wider AVF curve. This is indeed the observations from Fig. S16a.

Fig. S16b plots the spatial full width for 28 different crossings of LCC at different water depths. The expected trend from Eq. (10) agrees with the data points at 70–80 m but diverges at larger depths. To find the best-fitting trend of the data, we perform a linear regression and obtain a line with a slope of 0.66 and an intercept of 41.94. This indicates two different regimes, one for shallow water and one for deeper water. Unfortunately, water depths in the area range from 75 to 413 m. Therefore, we cannot analyse data points at water depths below 75 m. We speculate that our spherical movement assumption breaks down in deeper waters and that a non-spherical directivity pattern must be introduced. That said, the blue whales are within 40 m of the fibre, and the spherical assumption appears reasonable.

S13. What happens when the submerged object is close to the fibre?

When the moving object is close to the fibre, we need to examine the effect of the gauge length (L_g) on the recorded data. When the object is moving directly above the fibre ($y = 0$) the time strain is given as (from Eq.(10) main text):

$$\dot{\epsilon}_x = VR^2 \frac{z^2 - 2x^2}{(x^2 + z^2)^{5/2}}, \quad [14]$$

where V is the movement speed of the object, R is the radius of the inner boundary of the object and x, z are coordinates. To include the gauge length effect, we integrate Eq. (14) over one gauge length (rectangular gauge length window):

$$\dot{\epsilon}_{L_g} = \frac{1}{L} \int_{x-L_g/2}^{x+L_g/2} \frac{z^2 - 2\xi^2}{(\xi^2 + z^2)^{5/2}} d\xi, \quad [15]$$

$$\dot{\epsilon}_{L_g} = \frac{1}{2\gamma} \left(\frac{x+\gamma}{((x+\gamma)^2 + z^2)^{3/2}} - \frac{x-\gamma}{((x-\gamma)^2 + z^2)^{3/2}} \right) \quad [16]$$

where ξ is the integration variable, and we have introduced the gauge half-length as $\gamma = L_g/2$ to simplify the expression. Fig. S17 shows four examples of the gauge length effect where the gauge length is kept constant while the vertical distance from the fibre to the object is increasing. The main trend in the modelling is that the spatial full width, x_b, increases and the amplitude decreases as the vertical distance approaches the gauge length. As vertical distances increase, the gauge-length effects become smaller. Note that a rectangular window has been used here to represent gauge-length averaging for its simplicity. In practice, the window function varies across DAS systems, and the observed response varies accordingly.

S14. Distance decay of the hydrodynamic pressure signal

LCC sails along Adventfjorden, Isfjorden and Kongsfjorden several times during the cruise season. We leverage this data to identify times when the ship crosses the fibre at different positions and depths. This data is used to extract the amplitude for each crossing, assuming that the maximum amplitude occurs when it is directly above the fibre (see section S15). This results in Fig. S18 where the observed strain levels are plotted together with different decay functions. The observed curve agrees well with the expected $1/z^3$ curve (Eq (11) in the main text). The data in Fig. S18 are velocity corrected. This velocity correction is based on the different sailing speeds of LCC when it crosses the fibre, with a median sailing speed of 7 m/s, which is used as the reference value, V_{ref}. The following is used for the correction:

$$\epsilon_x' = \epsilon_x \frac{V_{ref}}{V} \quad [17]$$

where ϵ_x' is the corrected strain value, ϵ_x is the measured strain, and V is the individual sailing speed at the crossing time.

Some data points do not agree with the $1/z^3$ trend. This is the case when ships pass the fibre at shallow water depths and at low sailing speeds. Additionally, the data points at ~410 m have higher amplitudes than those at 320–340 m. We speculate that this is due to the angle at which the ship crosses the fibre, the DAS system's directivity and the local bathymetry profile.

S15. Strain rate model of LCC in the x–y plane

The strain rate model given in Eq. (10) of the main text is visualised in Fig. S19 based on the dimensions of Le Commandant Charcot. We can extract essential information from this figure: (1) The maximum amplitude occurs at the origin, i.e., directly under the vessel; (2) At a distance from the ship, the amplitude will flip sign. This agrees with the discussion made in "Materials and Methods" section the main text.

S16. Schematic illustration of the simple fluid model

To better understand the low-frequency data observed for whales and ships, we model responses using Rayleigh's work on the development of pressure during a cavity collapse in an incompressible liquid (16). Fig. S20 shows a schematic illustration of the problem for a single time instance together with the essential parameters presented in the method section of the main text. In Fig. S20a, we show the model for ship and whale examples. In Fig. S20b, we focus on the whale example and simplify the problem by equalising the whale movement to an oscillatory motion of a sphere. Let V and R be the velocity and the radius, respectively, of the inner boundary of the object being studied, at a time t, and v be the particle velocity at any distance r from the centre of the cavity (Fig. S20b). See "Materials and Methods" in the main text for the equations derived from the model.

S17. Aspect ratio of different whale species based on length and weight

We use the work by Lockyer (17) to create a table of aspect ratios for the different whale species presented in his work. The mass, m, of a whale is given as

$$m = \rho V = \rho \frac{\pi}{12} D^2 L \quad [18]$$

Table S1. Length, weight and aspect ratios of different whales derived from the work by Lockyer (17). The detection range is computed for the largest animal reported in (17), hence the maximum detection range is reported.

Name	Length (m)	Weight (Tons)	Aspect ratio (-)	Maxmum detection range (m)
Blue whale	28.5	162	0.163	40.0
Fin whale	24.8	84	0.145	35.1
Sei whale	18.1	31	0.141	28.2
Bryde whale	17.8	37	0.158	28.9
Humpback whale	16.3	79	0.264	32.4
Minke whale	10.2	11	0.199	21.6
Gray whale	14.8	39	0.214	28.3
Right whale	17.1	75	0.239	32.3
Sperm whale	18.0	69	0.213	32.2

where V is the body volume of the whale, D is the diameter of the body at the widest part, L is the body length (from snout tip to the notch between the tail flukes) and ρ is the density of the whale, assumed to be 1000 kg/m^3. We can further introduce the aspect ratio as

$$\alpha = \frac{D}{L} \quad [19]$$

and by combining Eq. (18) to Eq. (19) we find that

$$\alpha = \sqrt{\frac{12m}{\rho \pi L^3}}. \quad [20]$$

We extract the weights and lengths from Table 1 of Lockyer's work and compute the corresponding aspect ratios. The result for the blue whale is 0.163, and the aspect ratio for other whales can be found in Table S1. We can further use that the maximum of the strain rate data is given when the whale is directly above the fibre:

$$\dot{\epsilon}_{x,max} = \frac{vR^2}{z_{min}^3}, \quad [21]$$

where z_{min} is the minimum distance from the fibre to the whale generating the low-frequency signal, i.e., the detection range. If we assume that $R \approx 2dL$, where d is the diameter of the whale and use Eq. (19) we find:

$$z_{min} = \left(\frac{V 2 \alpha L^2}{\dot{\epsilon}_{x,min}} \right)^{1/3} \quad [22]$$

Assuming a constant swim speed, we can group the constants in Eq. (22) as $a = 2V/\dot{\epsilon}_{x,min}$

$$z_{min} = a \left(\alpha L^2 \right)^{1/3}. \quad [23]$$

Since we have estimated the detection range for blue whales, we can use it to find an estimation of the constant a.

$$a = \frac{z_{min}}{(\alpha L^2)^{1/3}} \quad [24]$$

Plugging in the values gives 7.85. We use the estimated a value and Eq. (23) to find the detection range for all whales in Table S1. The results are given in the last column of Table S1.

References

1. J Colen, EB Kolomeisky, Kelvin–froude wake patterns of a traveling pressure disturbance. *Eur. J. Mech.* **85**, 400–412 (2021).
2. GM Scarpa, et al., The effects of ship wakes in the Venice Lagoon and implications for the sustainability of shipping in coastal waters. *Sci. reports* **9**, 19014 (2019).
3. T Torsvik, T Soomere, I Didenkulova, A Sheremet, Identification of ship wake structures by a time–frequency method. *J. Fluid Mech.* **765**, 229–251 (2015).
4. CG David, V Roeber, N Goseberg, T Schlurmann, Generation and propagation of ship-borne waves-solutions from a boussinesq-type model. *Coast. Eng.* **127**, 170–187 (2017).
5. LD Talley, *Descriptive physical oceanography: an introduction.* (Academic press), (2011).
6. C Ma, et al., Wavelengths of the highest waves created by fast monohull ships or catamarans. *Ocean. Eng.* **113**, 208–214 (2016).
7. EF Williams, et al., Distributed sensing of microseisms and teleseisms with submarine dark fibers. *Nat. communications* **10**, 5778 (2019).
8. JN Newman, *Marine hydrodynamics.* (MIT press), (1977).

9. K Aki, P Richards, *Quantitative seismology.* (MIT Press), (2002).
10. RA Rørstadbotnen, M Landrø, On the variation of ocean surface gravity wave characteristics with wind speed and direction: A case study from offshore Svalbard, Norway. *Geophys. J. Int.* p. ggaf240 (2025).
11. K Taweesintananon, et al., Near-surface characterization using shear-wave resonances: A case study from offshore Svalbard, Norway. *Geophysics* **89**, B317–B327 (2024).
12. CC Mei, MA Stiassnie, DKP Yue, *Theory and applications of ocean surface waves: Part 1: linear aspects.* (World Scientific), (2005).
13. M Landrø, et al., Sensing whales, storms, ships and earthquakes using an Arctic fibre optic cable. *Sci. Reports* **12**, 1–10 (2022).
14. L Bouffaut, et al., Eavesdropping at the speed of light: Distributed acoustic sensing of baleen whales in the Arctic. *Front. Mar. Sci.* **9**, 901348 (2022).
15. kystdatahuset.no, Toposvalbard - Norsk polarinstitutt (2025) Accessed: 2025-09-30.
16. L Rayleigh, VIII. On the pressure developed in a liquid during the collapse of a spherical cavity. *The London, Edinburgh, Dublin Philos. Mag. J. Sci.* **34**, 94–98 (1917).
17. C Lockyer, Body weights of some species of large whales. *ICES J. Mar. Sci.* **36**, 259–273 (1976).

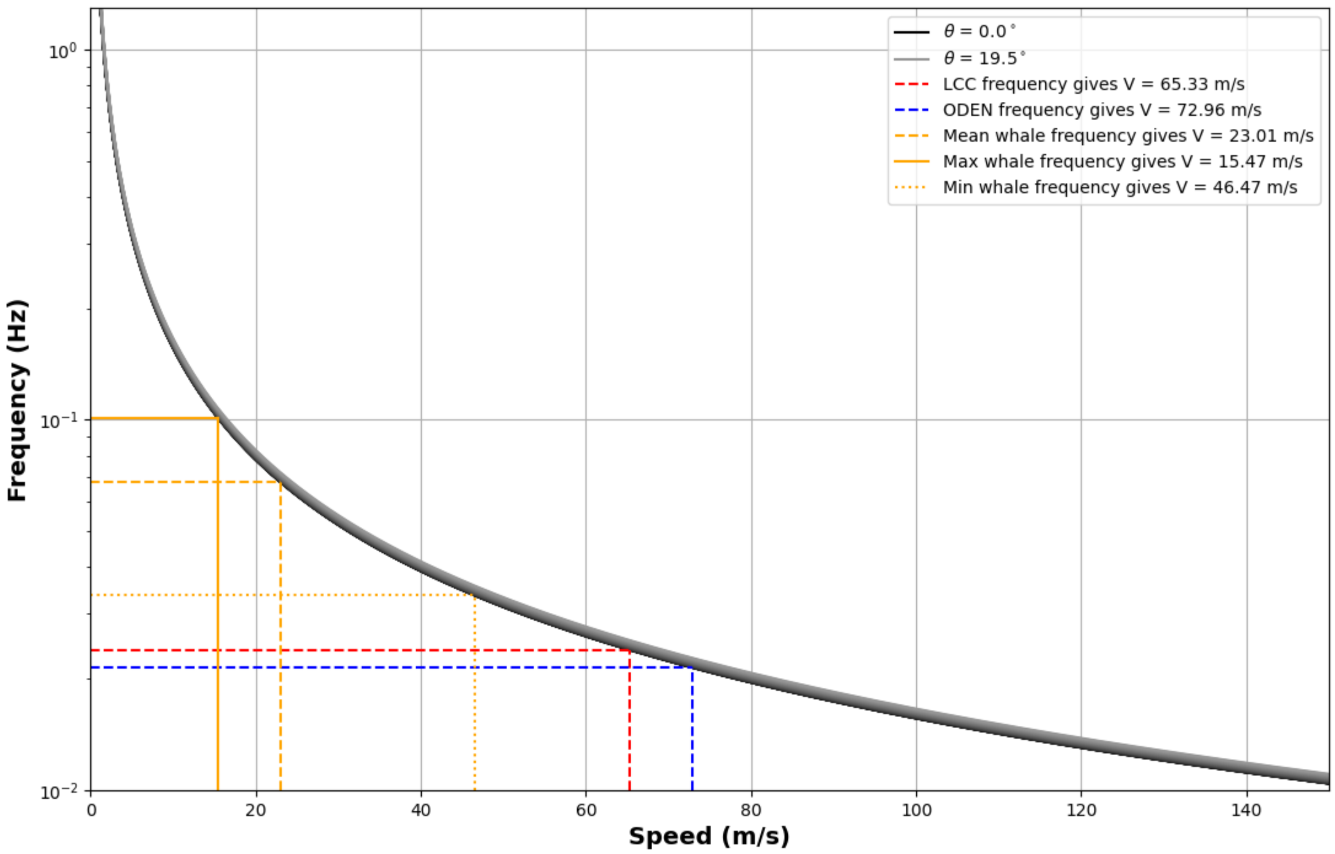

Fig. S1. Phase velocity – frequency relation from Kelvin wake theory. Curves are given for different angles θ from black ($\theta = 0$) to grey ($\theta = 19.5$). The speeds needed to obtain the various observed frequencies are depicted as dashed, dotted, and solid lines.

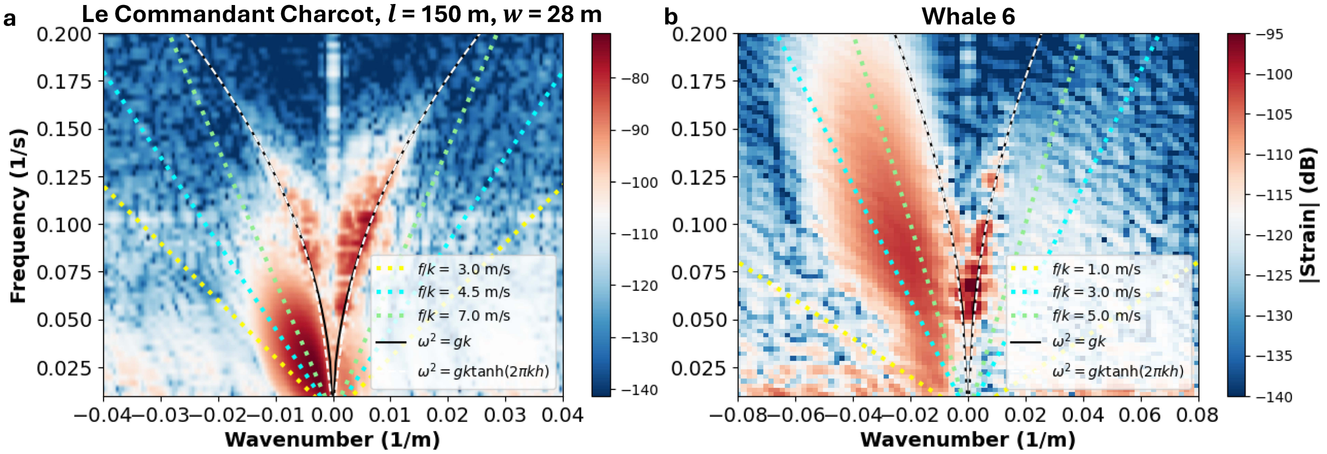

Fig. S2. Comparison between LCC and whale 6 frequency-wavenumber domain representation. (a) Frequency-wavenumber domain representation of LCC. (b) Frequency-wavenumber domain representation of whale 6. Dashed lines indicate the dispersion relation for ocean surface gravity waves and the relevant movement speeds. Note the difference in the x–axis, meant to focus on the main energy of the vessel and the whale.

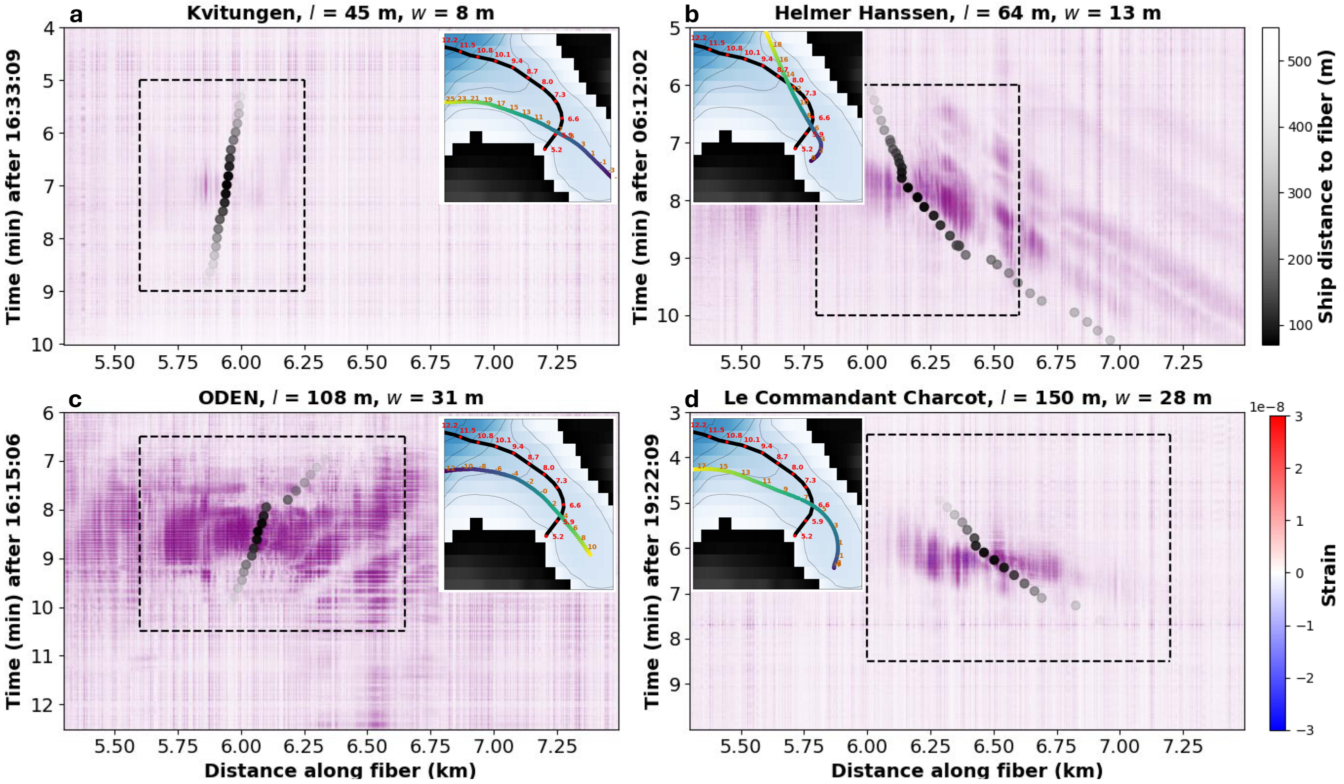

Fig. S3. Acoustic, higher-frequency, signatures of four ships of different sizes in the time-distance domain. a) The smallest ship, Kvitungen, with a length of 45 m and a width of 8 m. b) The ship Helmer Hansse with a length of 64 m and a width of 13 m. c) The icebreaker ODEN with length 108 m and width 31 m. d) The largest ship, the cruise ship Le Commandant Charcot, length 150 m and width 28 m. In all figures, the distance from the AIS position to the nearest virtual DAS channel is indicated by grey circles, with transparency decreasing with decreasing distance. The insets show the AIS track relative to the fibre cable, and dashed rectangles indicate the windows used for frequency and frequency-wavenumber analysis.

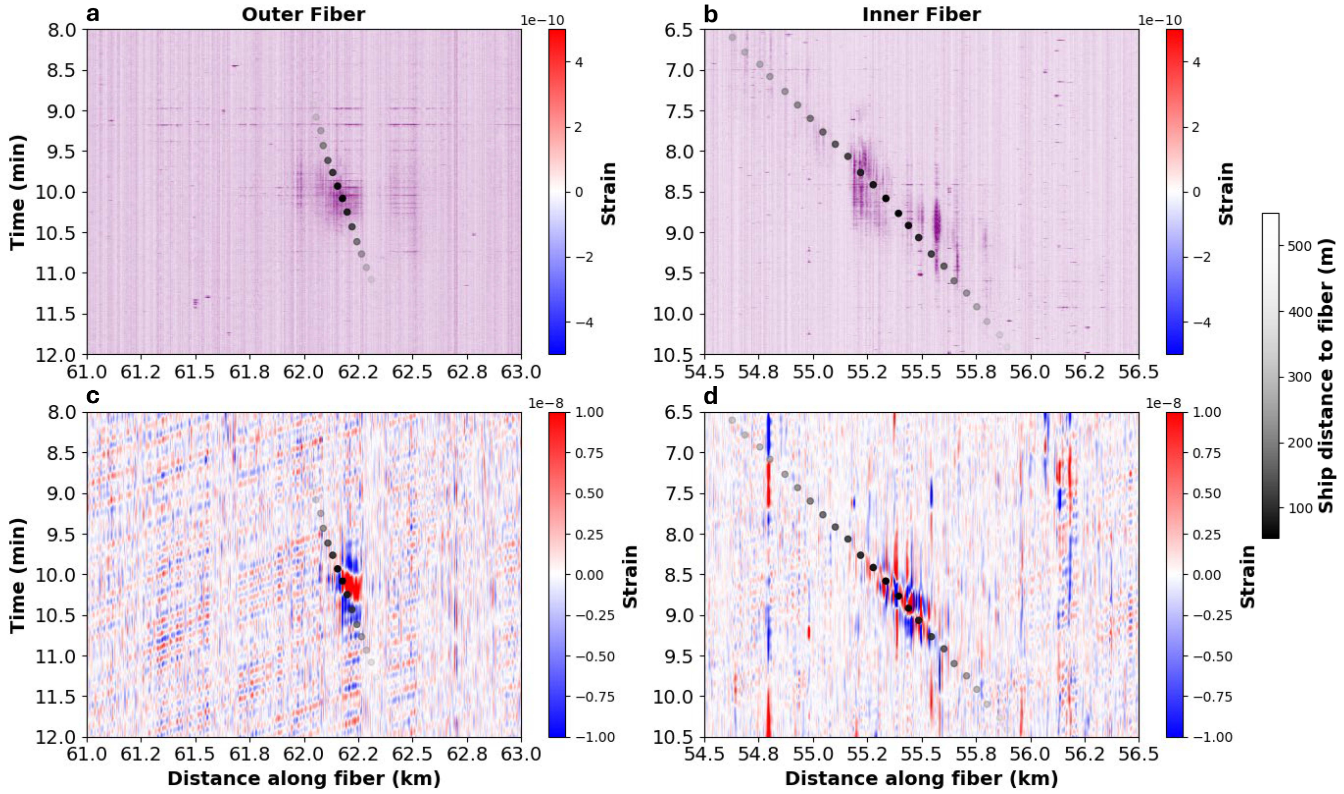

Fig. S4. LCC recorded on unburied sections of the inner and outer fibre. (a) The acoustic signature for the outer fibre. (b) The acoustic signature for the inner fibre. (c) The hydrodynamic pressure signal for the outer fibre. (d) The hydrodynamic pressure signal for the inner fibre.

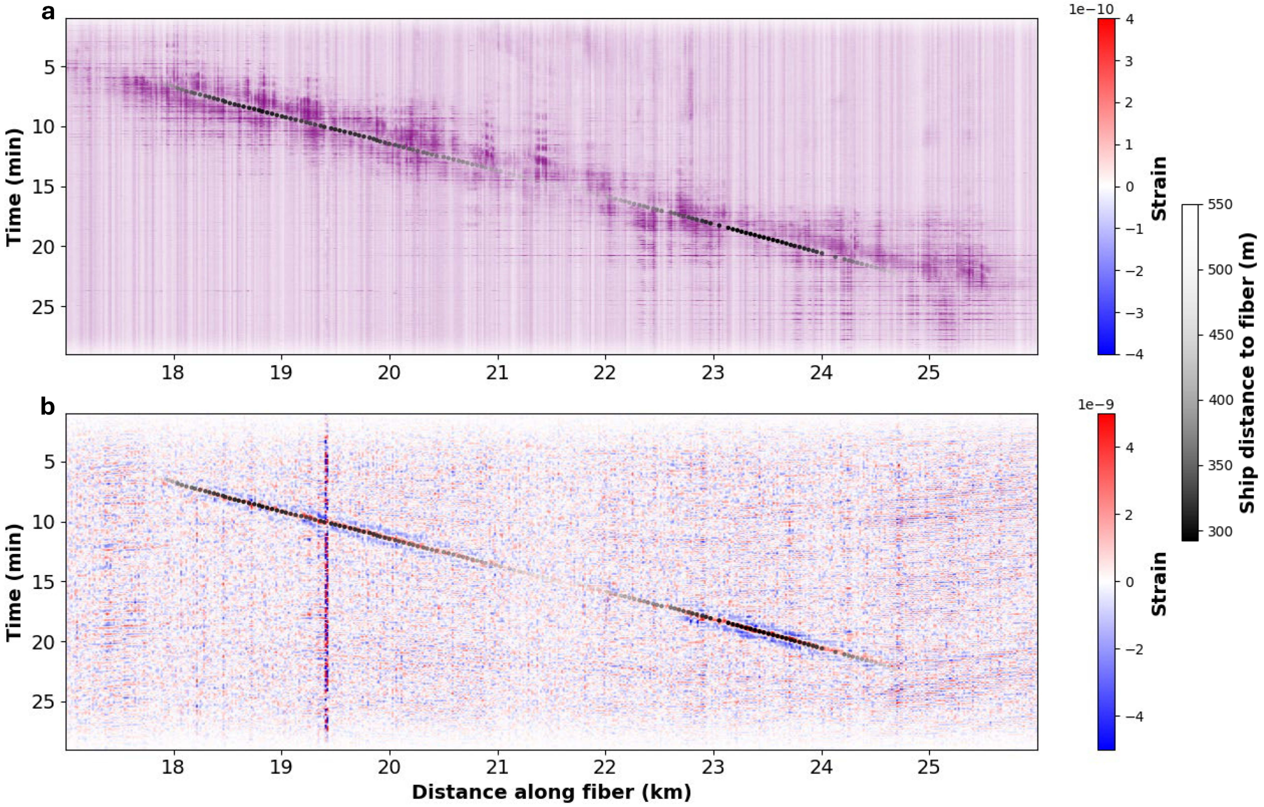

Fig. S5. LCC recorded on a 7 km section of buried inner fibre in Kongsfjorden. (a) The acoustic signature. (b) The hydrodynamic pressure signal.

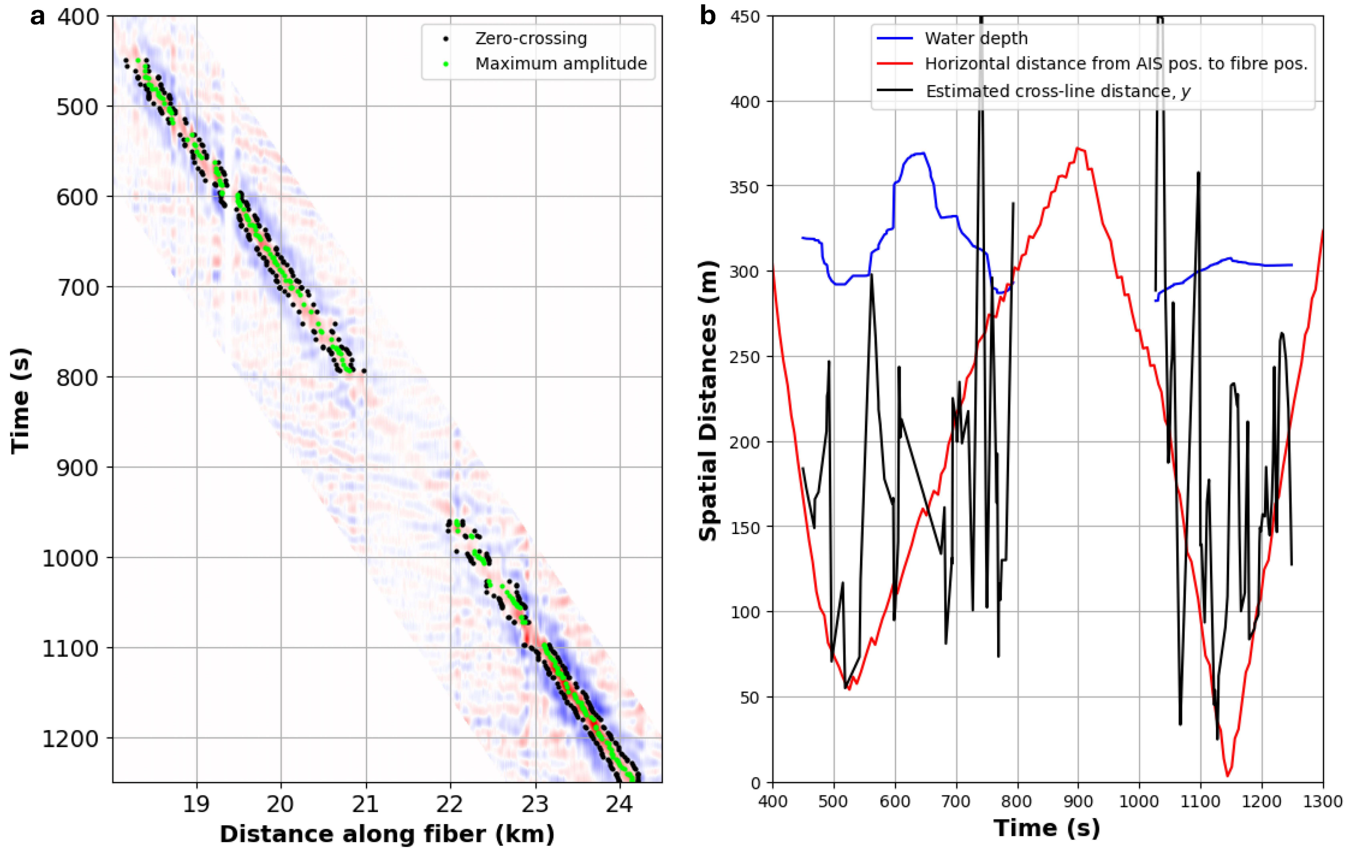

Fig. S6. Estimation of cross-line distance, y, from fibre to vessel position. (a) t–x showing how the zero-crossing, x_b, is extracted. For time samples, we extract the maximum value (green dot) and the closest zero-crossing on each side (see Figure S7 for waveform examples). Note that channels do not have repeated entries. (b) The resulting cross-line distance, y (black), compared to the horizontal distance from AIS (red) and the water depth in the points used (blue).

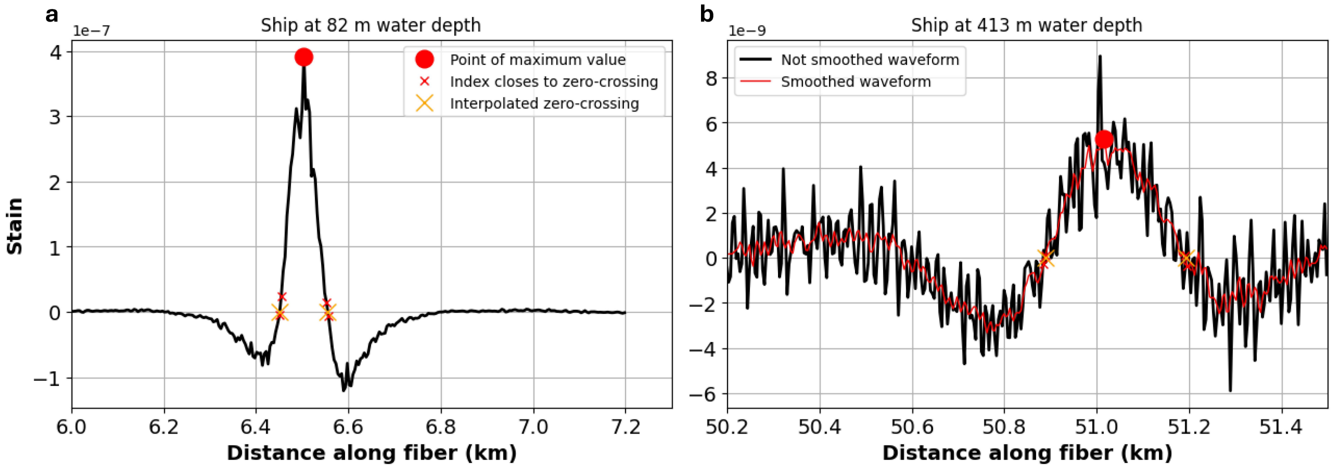

Fig. S7. Waveform examples showing the distance estimation between zero-crossings adjacent to the maximum value. (a) Maximum amplitude waveform where LCC crosses 82 m above the fibre. (b) Maximum amplitude waveform for a crossing 413 m over the fibre.

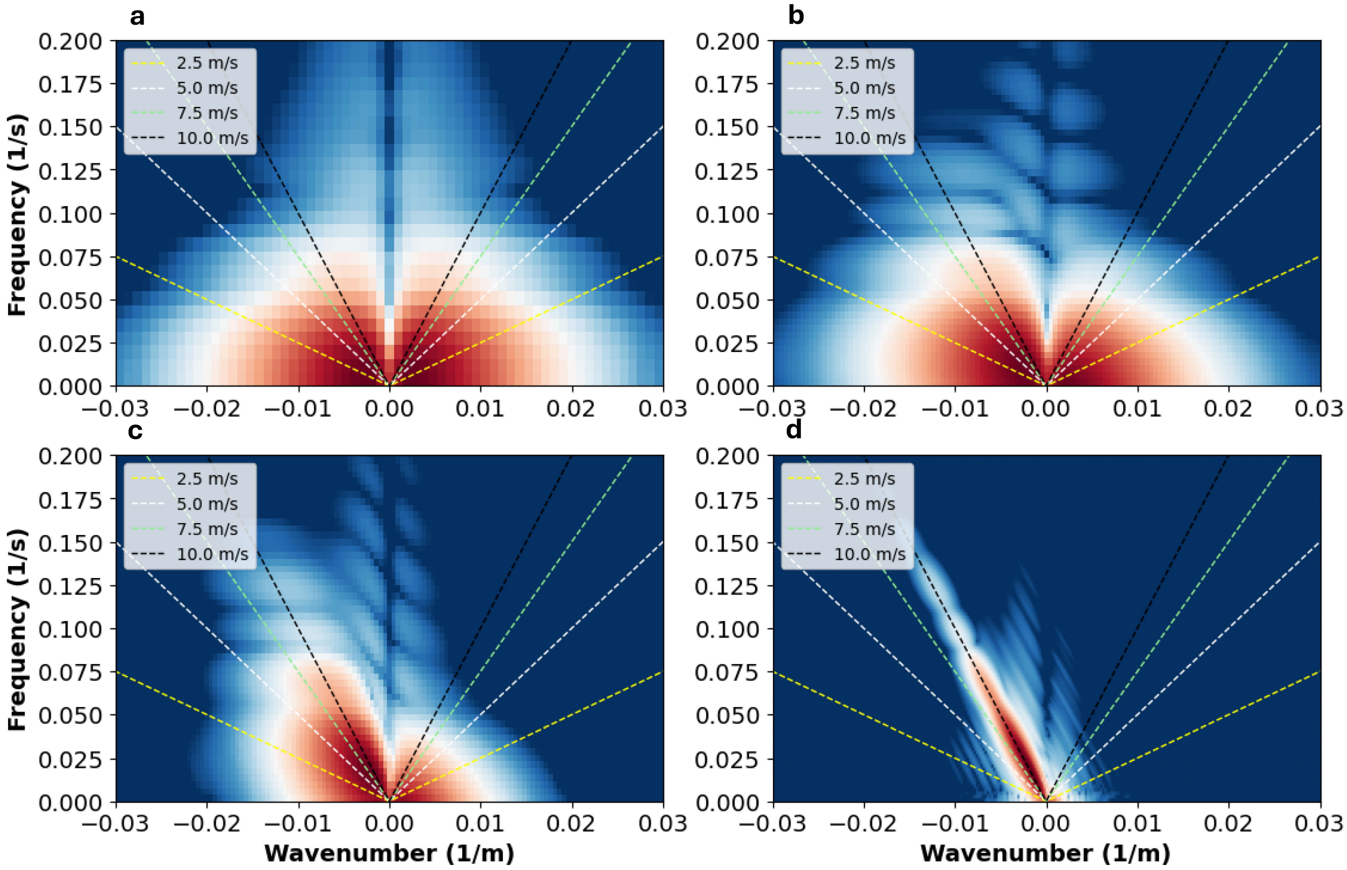

Fig. S8. Hydrodynamic modelling results in the frequency-wavenumber domain. Modelled f–κ response using a sinusoidal wavelet as source and the dimensions of LCC. (a) Ship crosses the fibre perfectly perpendicular (90°) to the fibre. (b) Ship crosses at 78.1°. (c) Ship crosses at 53.1°. (d) Ship crosses at 8.1°. Note that ship paths relative to the fibre cable are depicted in Fig. 3 of the main text.

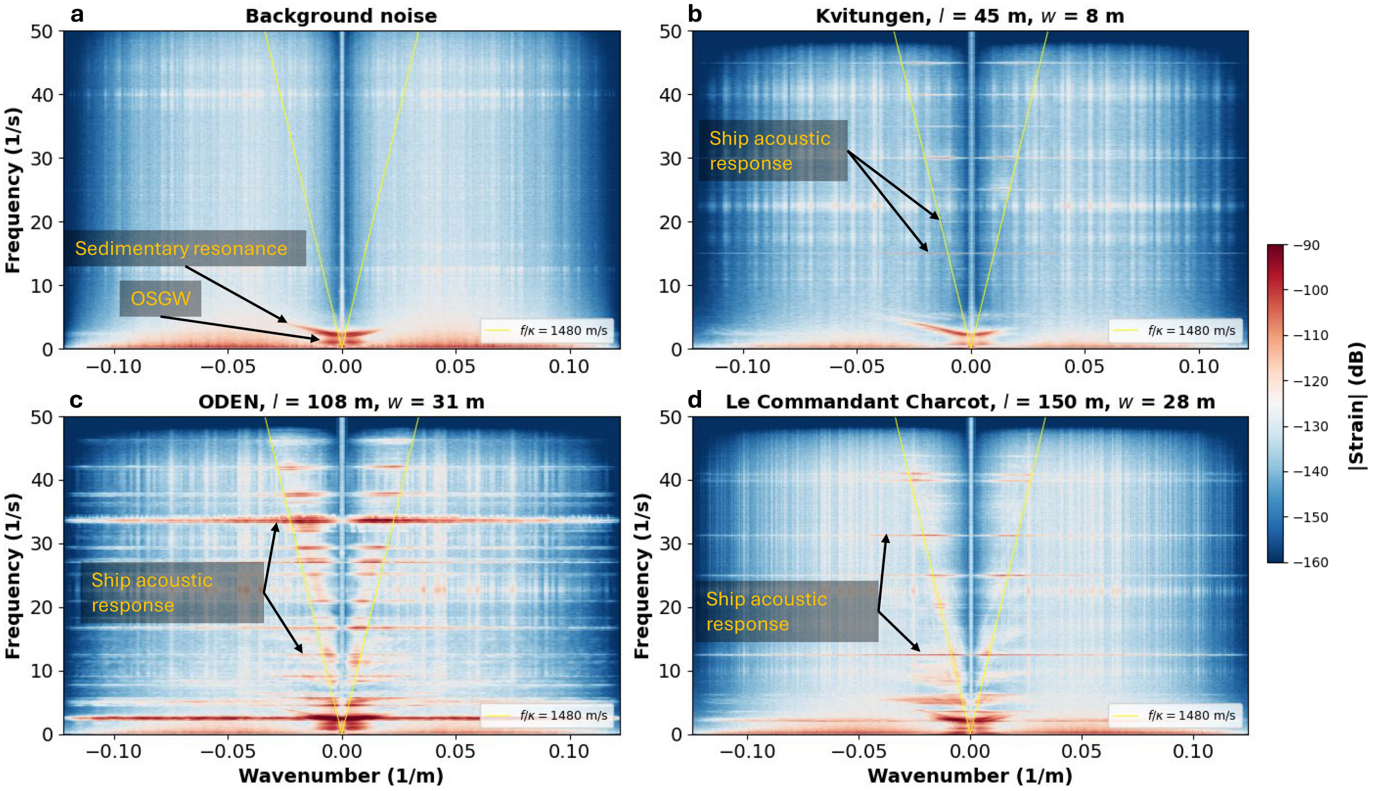

Fig. S9. High-frequency signatures of four ships of different sizes in the frequency-wavenumber domain. The yellow line in each panel shows the expected frequency wavenumber pair for an acoustic pressure wave propagating with 1480 m/s. Annotations indicate the various responses in the data and their interpreted origin.

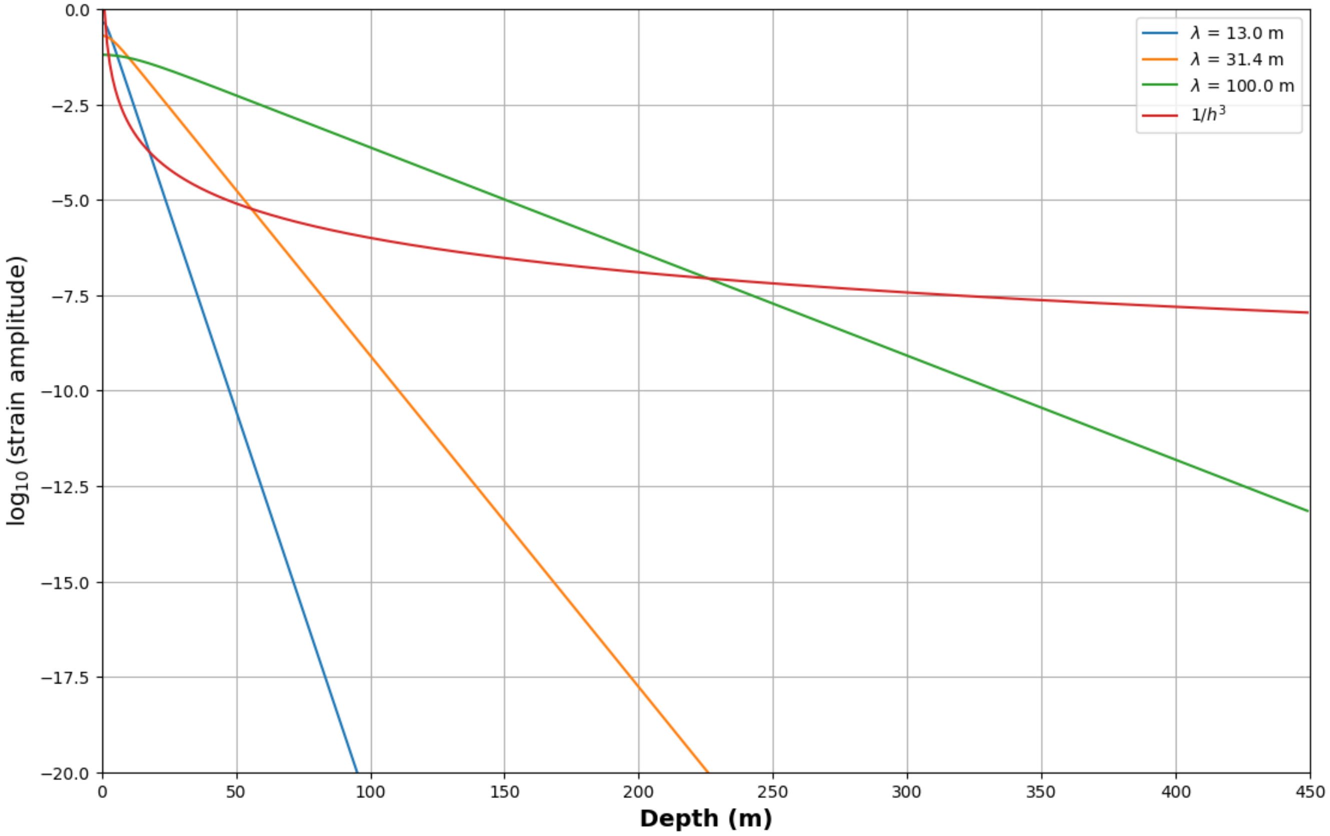

Fig. S10. Expected delay of strain amplitude with depth for ocean surface gravity waves of different wavelengths (blue, orange, green) compared to the observed $1/h^3$ trend for the hydrodynamic pressure signals.

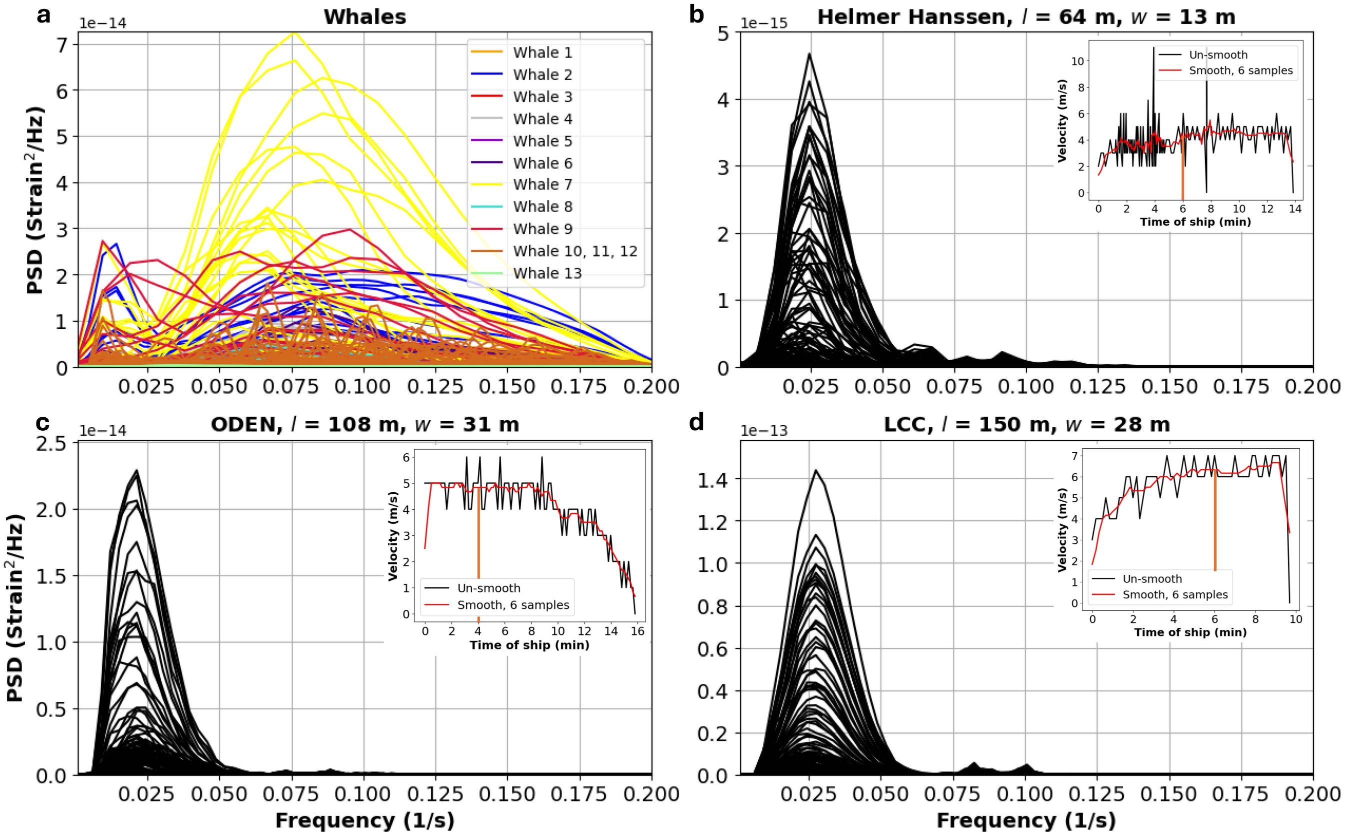

Fig. S11. Frequency content of all blue whales, HH, ODEN and LCC. (a) Frequency of different whale signals. (b) Frequency of HH. (c) Frequency of ODEN. (d) Frequency of LCC. The inset shows the speed estimates from the AIS, with the vertical orange line indicating the ship-fibre cross time.

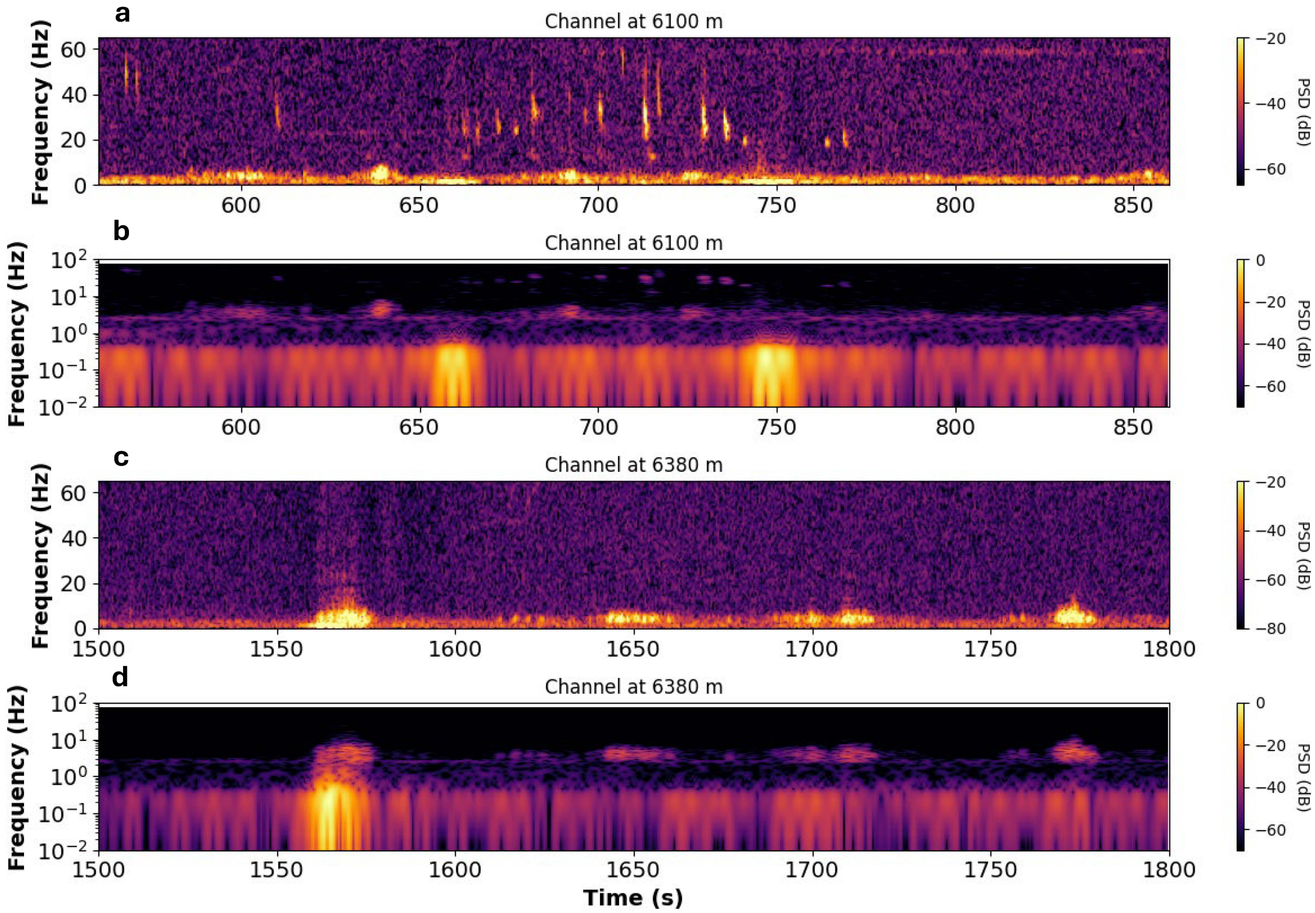

Fig. S12. Spectrograms identifying acoustic vocalisations, Scholte waves and hydrodynamic pressure signals from blue whales. (a) High-frequency data from the channel at 6.1 km capturing acoustic signals from whales. (b) Low-frequency data from the channel at 6.1 m capturing Scholte waves and hydrodynamic pressure signals. The data in (a) and (b) show how two hydrodynamic pressure signals envelope the blue whale downsweeps occurring between 660 s and 7550 s. (c) High-frequency data from the channel at 6.38 km. (d) Low-frequency data from the channel at 6.38 km. The data in (c) and (d) show examples where the hydrodynamic pressure data coincide with Scholte waves (1570 s) and other instances where they do not (e.g., around 1650 and 1700 s).

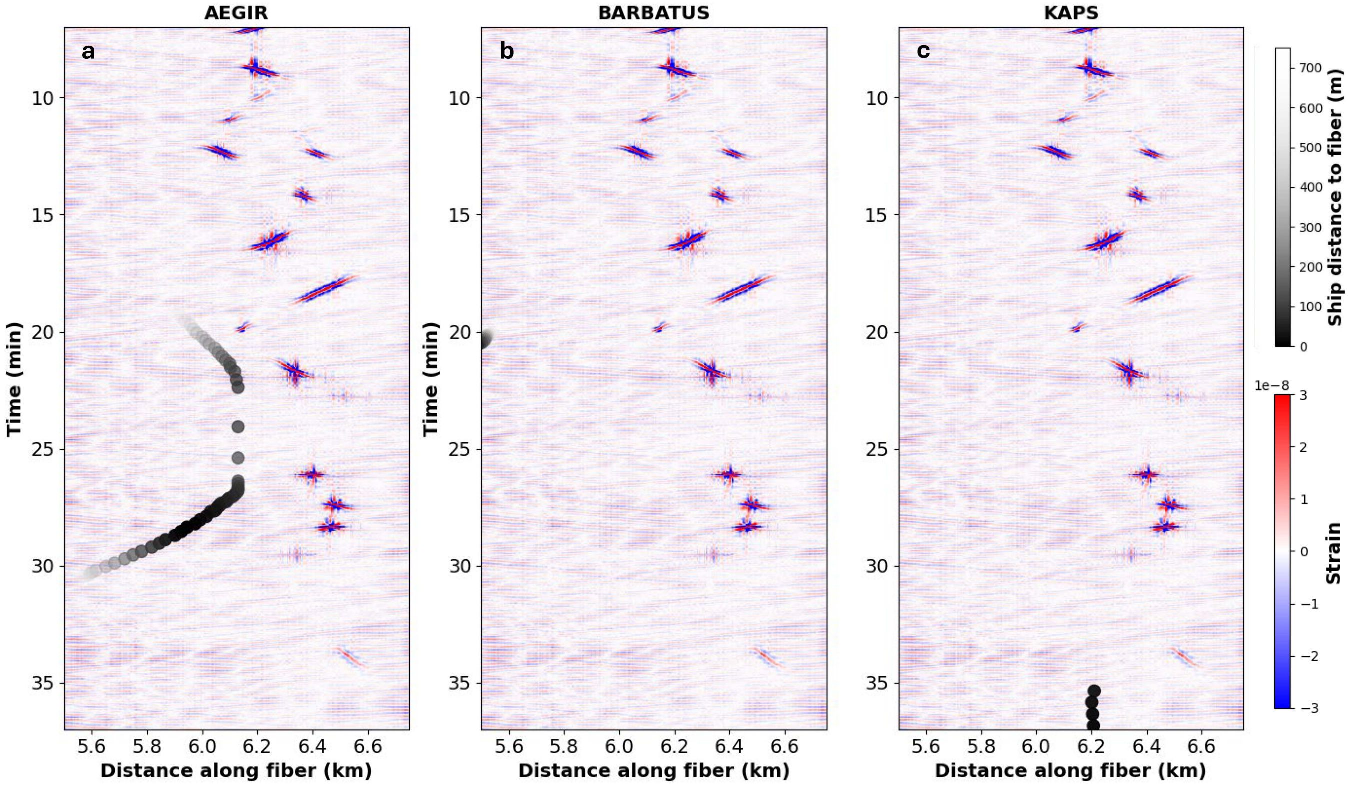

Fig. S13. Ships present in AIS simultaneous to whale vocalisation. (a) The AIS track of AEGIR is overlaid on the data. (b) The track of BARBATUS is overlaid. (c) The track of KAPS is overlaid. None of the ships overlaps with the observed signal.

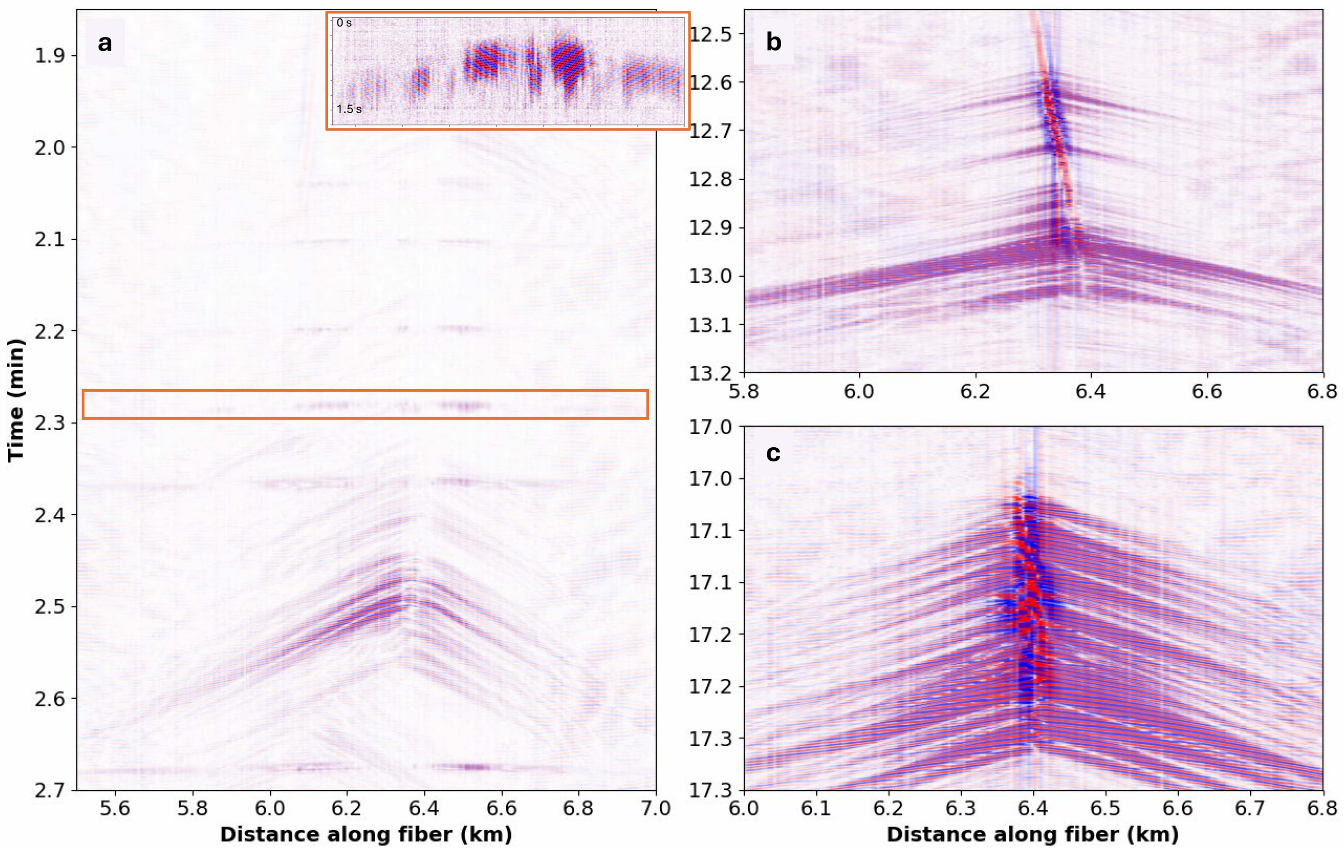

Fig. S14. High-frequency signatures overlaid transparently on the low-frequency signatures. (a) Blue whale downsweeps followed by Scholte wave. One set of hydrodynamic pressure signals is observed, but disconnected from the whale calls and Scholte wave. The orange inset zooms in on a blue whale call (note that the inset time scale is compressed). (b), (c) Two examples of Scholte wave overlaying hydrodynamic pressure signals perfectly.

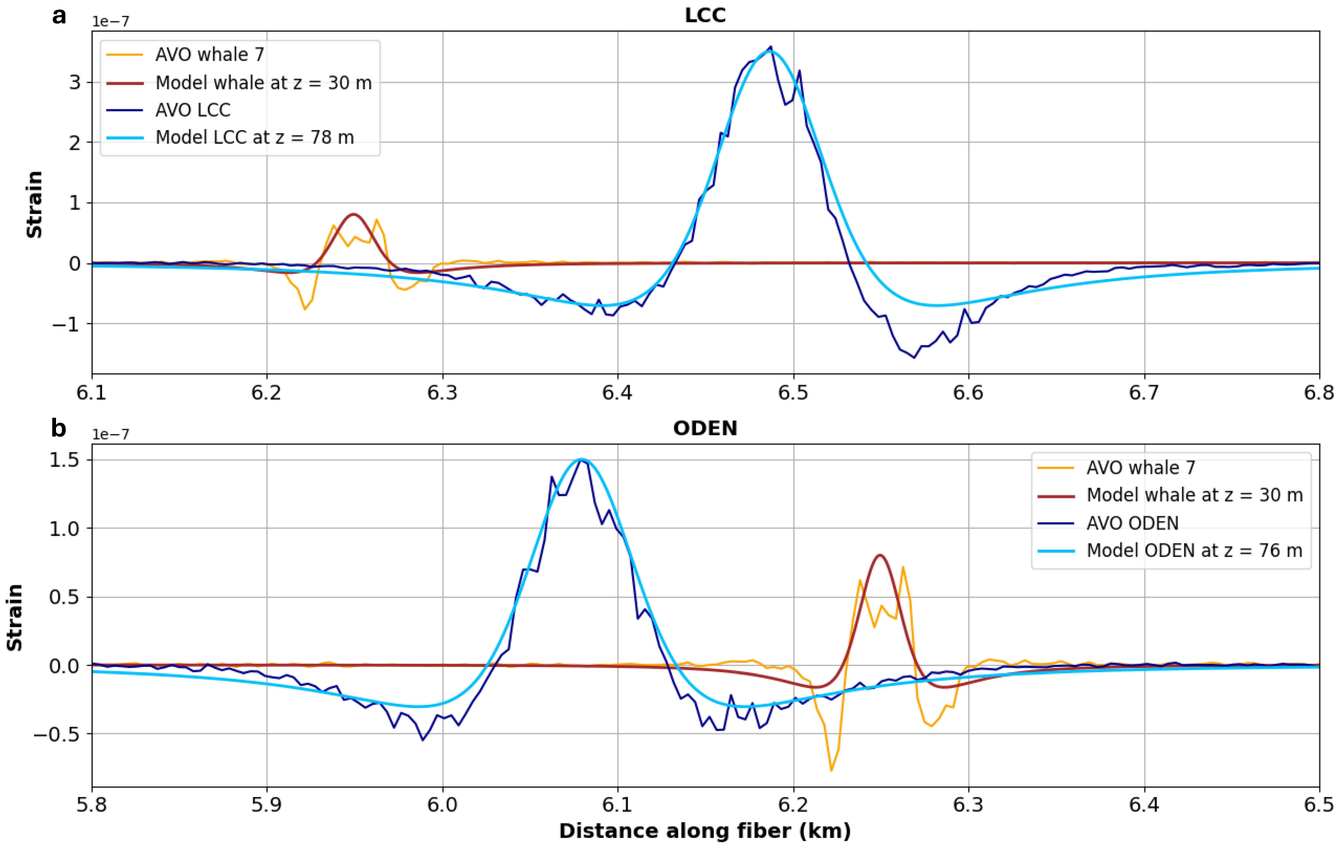

Fig. S15. Amplitude variation with offset for ships and whales. (a) Amplitude from LCC, a blue whale and the simple fluid-flow model. (b) Amplitude from ODEN, a blue whale and the simple fluid-flow model. The models are generated using Eq. (10) in the main text. The water depth where the ships sailed at the time of recording, and the estimated blue whale depth (30 m) are used. Note that the ships and whale are not recorded at the same time; they are plotted together.

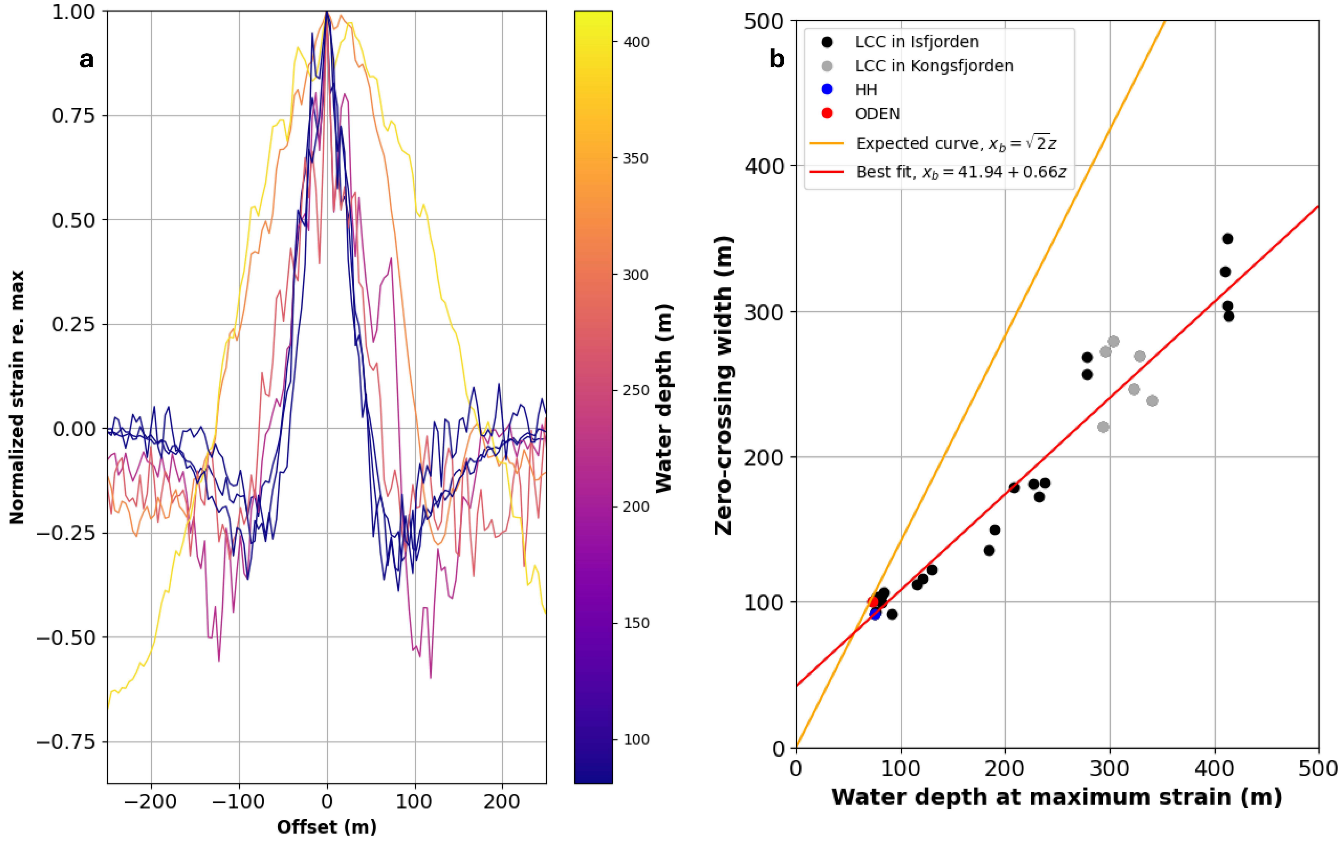

Fig. S16. Amplitude-along fibre (AVF) response for LCC sailing at different water depths. (a) AVF curves colour-coded by water depth, normalised relative to maximum, to focus the comparison on the increasing spatial pulse width with increasing distance from the vessel to the fibre position. Note that the AVF curves for when LCC sailed at 280 m and 407 m water depth were smoothed for more reliable zero-crossing picking (see Figure S7). (b) Expected curve from theory given in the main text, the DAS data points and the best fitting curve. Note the change in slope at 80 m, indicating the need for further work on the theory.

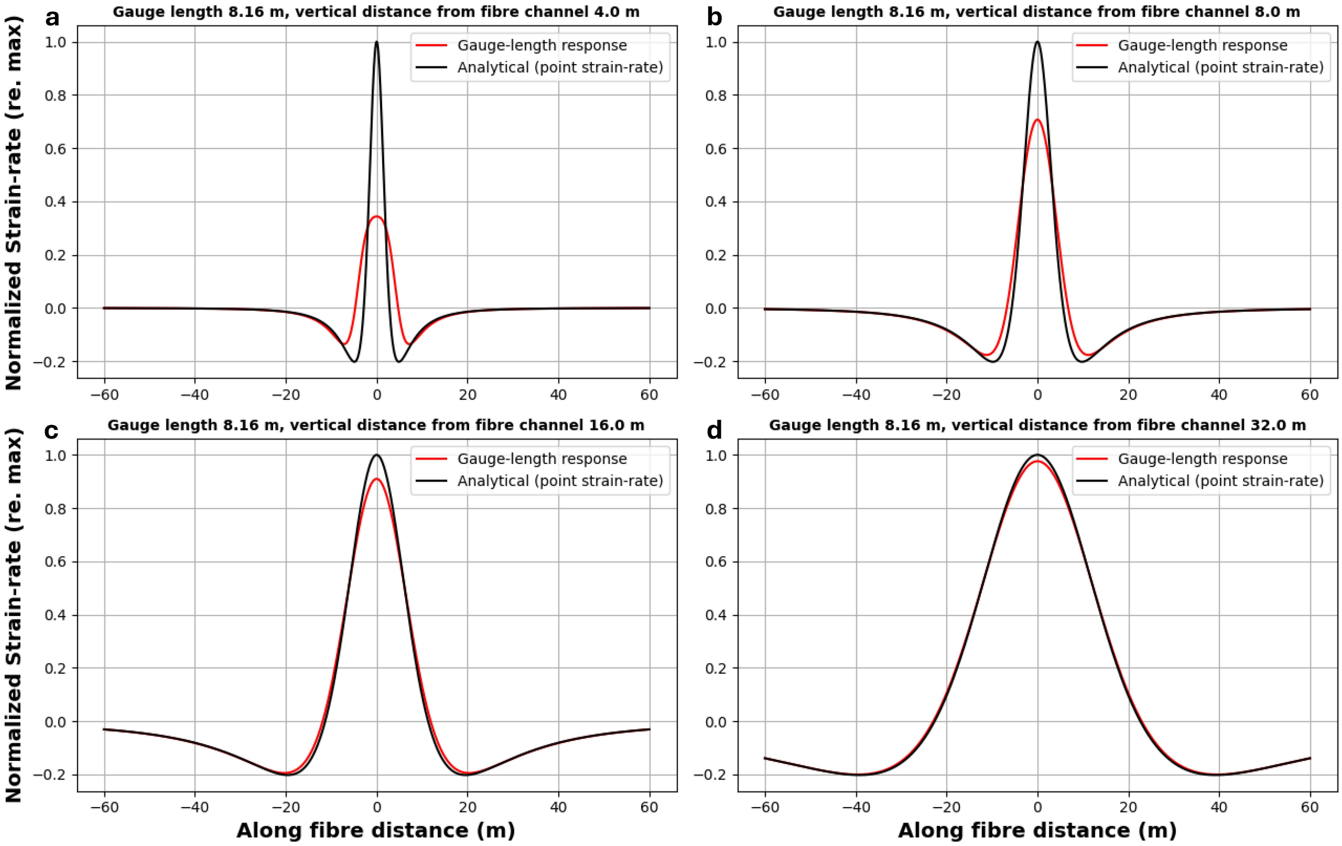

Fig. S17. Gauge length response using a rectangular averaging window at increasing distances from the fibre. Gauge length of 8.16 m used. (a) Distance equal to roughly half the gauge length, $z = 4$ m. (b) Distance roughly equal to the gauge length, $z = 8$ m. (c) Distance equals 16 m. (d) Distance equal to 32 m.

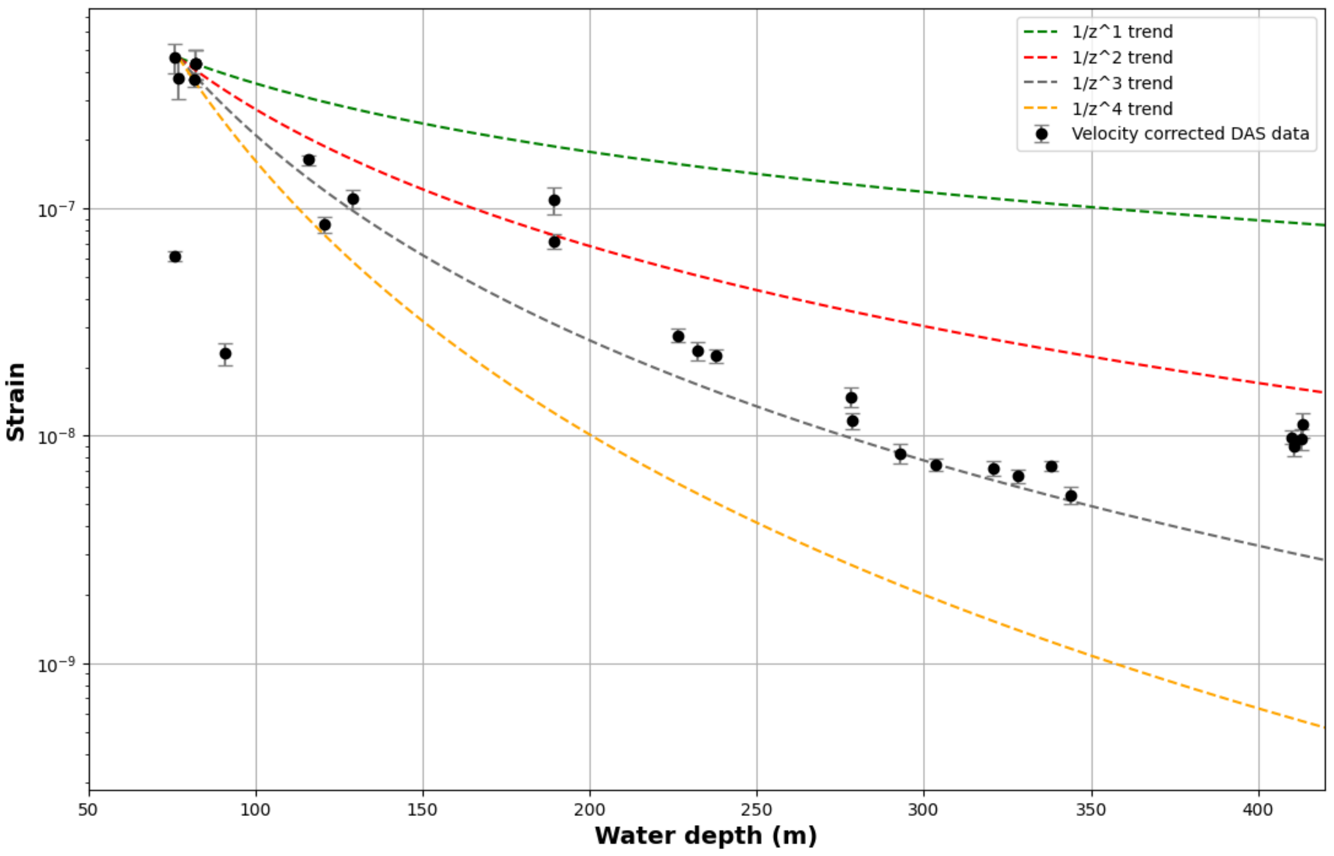

Fig. S18. Signal decay of the hydrodynamic pressure signals as observed using DAS. The error bars are the mean and one standard deviation of the top 1% of strain amplitudes. The main trend follows the $1/z^3$ curve, with outliers at shallow and deep water depths.

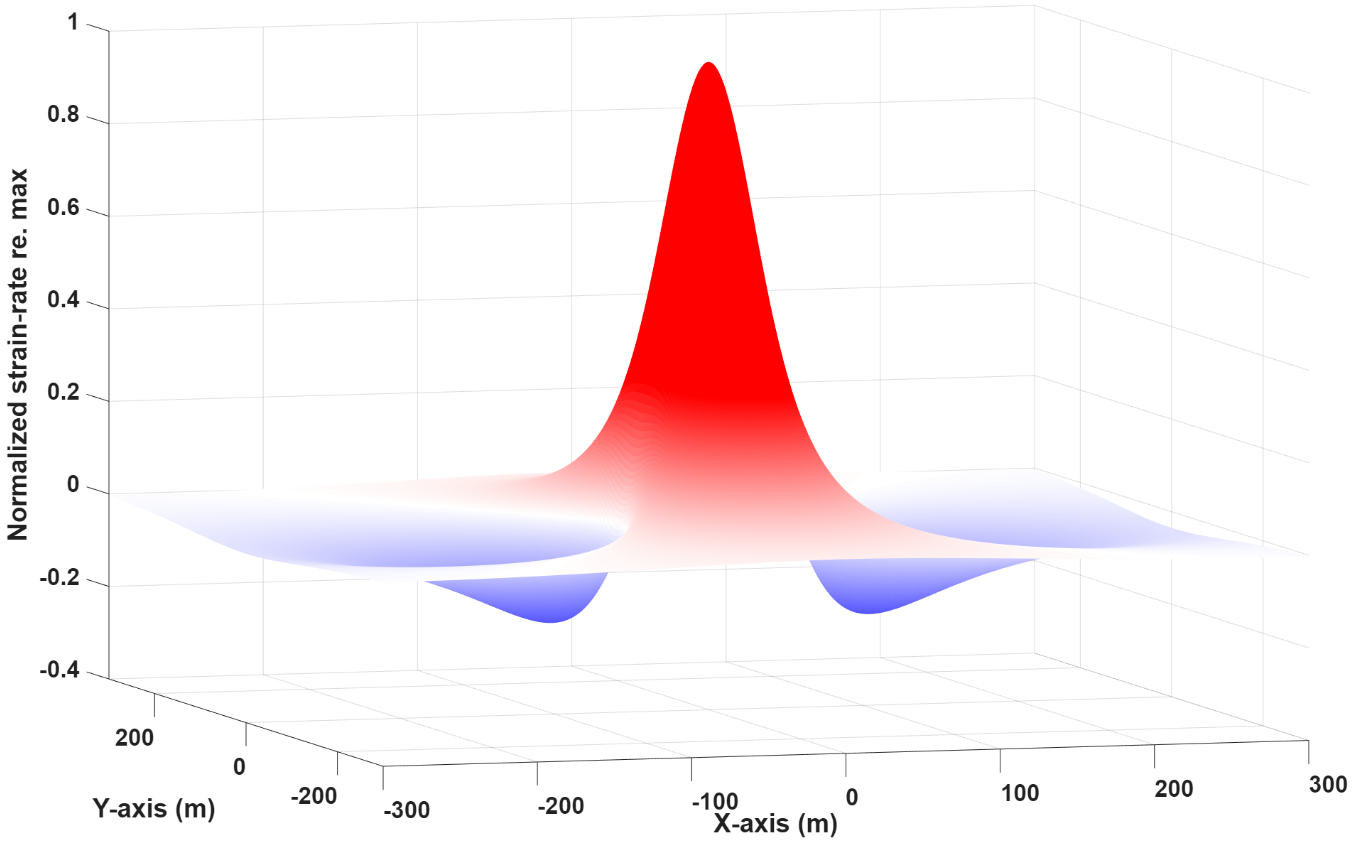

Fig. S19. The strain response in 3-dimensions from Eq (10).

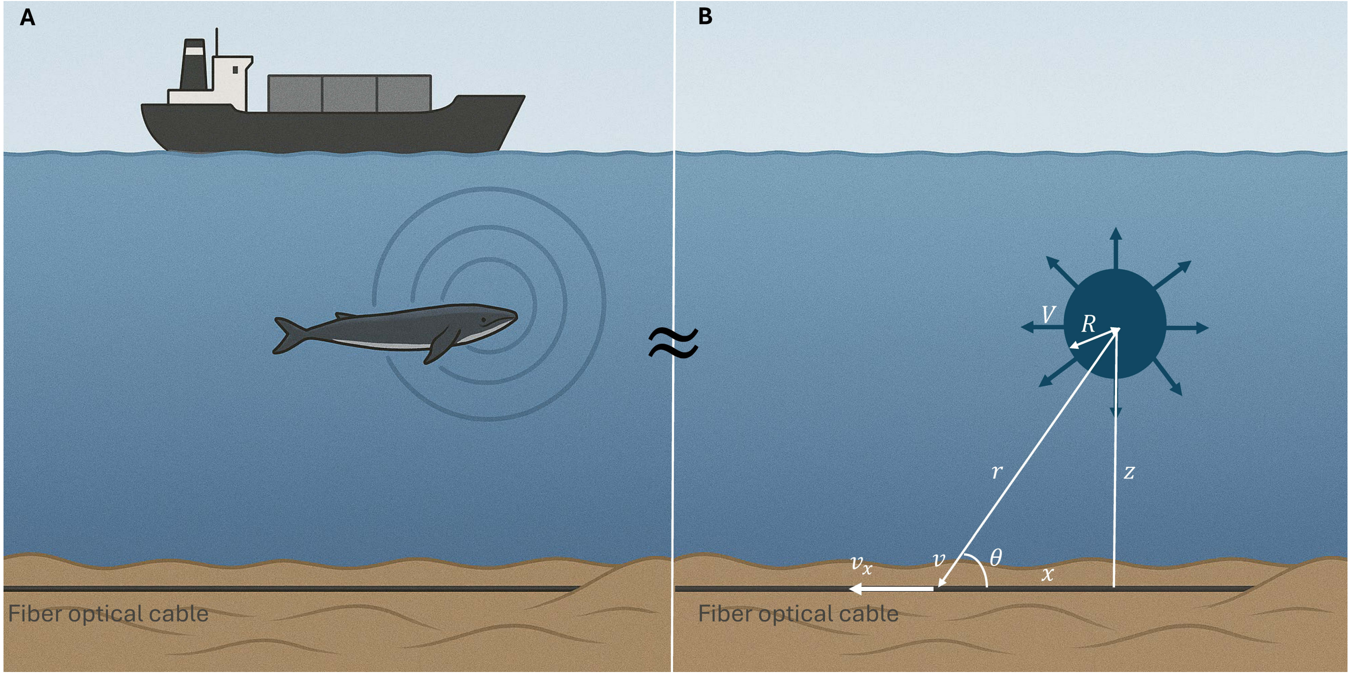

Fig. S20. Schematic illustration of the physical concept used in the modelling. (a) Illustration of the actual scenario. A whale (or a ship) swims (or sails) across the ocean. They will generate acoustic signals (e.g., vocalisation or propeller noise) that propagate to the fibre and be recorded. In addition, they displace water, causing pressure fluctuations that propagate to the seafloor sediments and the fibre and deform them. (b) Our simplification of the problem for low-frequency signals is illustrated for the whale case. The whale is assumed, as a first-order assumption, to be a sphere that displaces water. This sphere has a radius of R, moves with a speed V and generates a change in particle velocity v at a distance r from the centre of the circle. We record the in-line component, which we assume coincides with the x-axis, hence v_x. Note that only the geometrical shape of the whale is depicted in (b). See 'Materials and Methods' for a detailed description of the assumed geometrical shapes.
